# Magnetic resonance imaging of brain cell water

**DOI:** 10.1038/s41598-019-41587-2

**Published:** 2019-03-25

**Authors:** Takashi Watanabe, Xiaoqing Wang, Zhengguo Tan, Jens Frahm

**Affiliations:** Biomedizinische NMR, Max-Planck-Institut für biophysikalische Chemie, Göttingen, Germany

## Abstract

In the central nervous system of vertebrates, cell bodies of neurons are often assembled as nuclei or cellular layers that play specific roles as functional units. The purpose of this work was to selectively highlight such cell assemblies by magnetic resonance imaging using signals from water protons that are associated with intracellular paramagnetic ions, while saturating lipid-associated water protons as well as extracellular free water protons. Given the significant correlation between image signal intensity and water proton density, the high signal intensities observed for such cell assemblies must be attributed to their abundant paramagnetic-ion-associated water protons. In the hippocampal formation, the technique visualized cell assemblies that were so far not depicted in human *in vivo*. In the brainstem, the technique delineated noradrenergic neuron groups such as the locus coeruleus in human and mice *in vivo*. Their reduced magnetization-transfer ratios together with their prolonged relaxation times compared to other gray matter indicate that the source of their high signal intensity is not the presence of T_1_-shortening molecules, e.g., neuromelanin, but their high water content. Given the general absence of neuromelanin in noradrenergic neurons of rodents, their high signal intensity in mice *in vivo* further supports this view.

## Introduction

**Figure 1:**
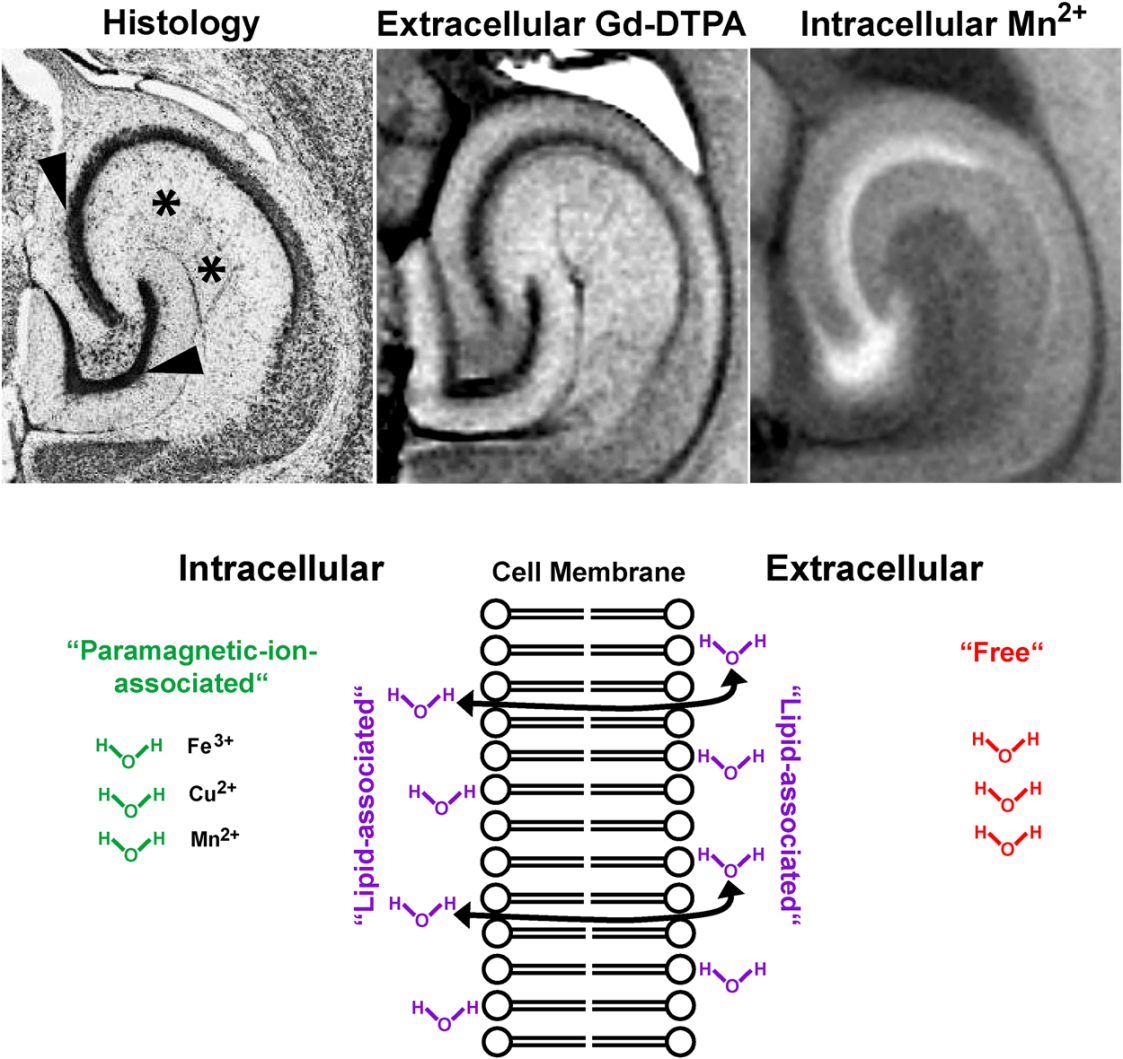
(Top) (left) Nissl stain and (middle) T_1_-weighted MR images of the hippocampal formation of mice *in vivo* (9.4T, spoiled 3D FLASH, TR/TE 22/7.6 ms, flip angle α 25°, 25 × 25 × 250 µm^3^ resolution) after intraventricular injection of gadolinium-diethylenetriamine pentaacetic acid (Gd-DTPA), and (right) after systemic administration of manganese (Adapted from^3,49^). After Gd-DTPA the signal intensities in the cellular layer are lower than in the neuropil (and higher after Mn^2+^) indicating that the cellular layer (arrowheads) has a lower content of extracellular water protons than the neuropil (asterisks). (Bottom) Schematic drawing of three major brain water pools that contribute to MRI: “Lipid-associated” (purple) water protons which may be affected by cross-relaxation or magnetization transfer, “paramagnetic-ion-associated” (green) water protons in intracellular spaces, and “free” (red) bulk water protons in extracellular spaces.

In the central nervous system of vertebrates, cell bodies of neurons are often assembled as nuclei or cellular layers that play specific roles as functional units, e.g. in the hippocampal formation as shown in Fig. 1 (top left, arrowheads). Magnetic resonance imaging (MRI) can readily differentiate large cell assemblies from surrounding tissue. This is because intracellular water protons exhibit T_1_ and T_2_ relaxation times which are generally smaller than those of bulk water protons in extracellular spaces^1^ (denoted “Free“ in Fig. 1), but larger than those of (intra- and extra-cellular) water protons that interact with lipid bilayers^2^ (denoted “Lipid-associated“ in Fig. 1). After administration of exogenous contrast agents^3^, also small cell assemblies in the hippocampal formation (Fig. 1, top left, arrowheads) can be identified and distinguished from the neuropil (Fig. 1, top left, asterisks) and spaces filled with cerebrospinal fluid (CSF).

Here we attempt to gain signals selectively from the water protons that are associated with intracellular paramagnetic ions by saturating extracellular free water protons by direct on-resonance irradiation, as well as by eliminating signal contributions from lipid-associated protons by off-resonance irradiation of short T_2_ lipid protons with broad linewidths. The latter method indirectly saturates lipid-associated water protons through magnetization transfer^4^ (MT). The concept neither runs counter to previous studies showing fast water exchange between extra- and intra-cellular spaces relative to T_1_ relaxation times^1^ nor attempts to differentiate between extra- and intra-cellular pools of water protons. Instead, we propose a new strategy to preferentially image the fraction of intracellular water protons (“Paramagnetic-ion-associated“ in Fig. 1).

## Results

### Magnetization-transfer MRI of the brain provides proton-density contrast while saturating extracellular free water protons

**Figure 2:**
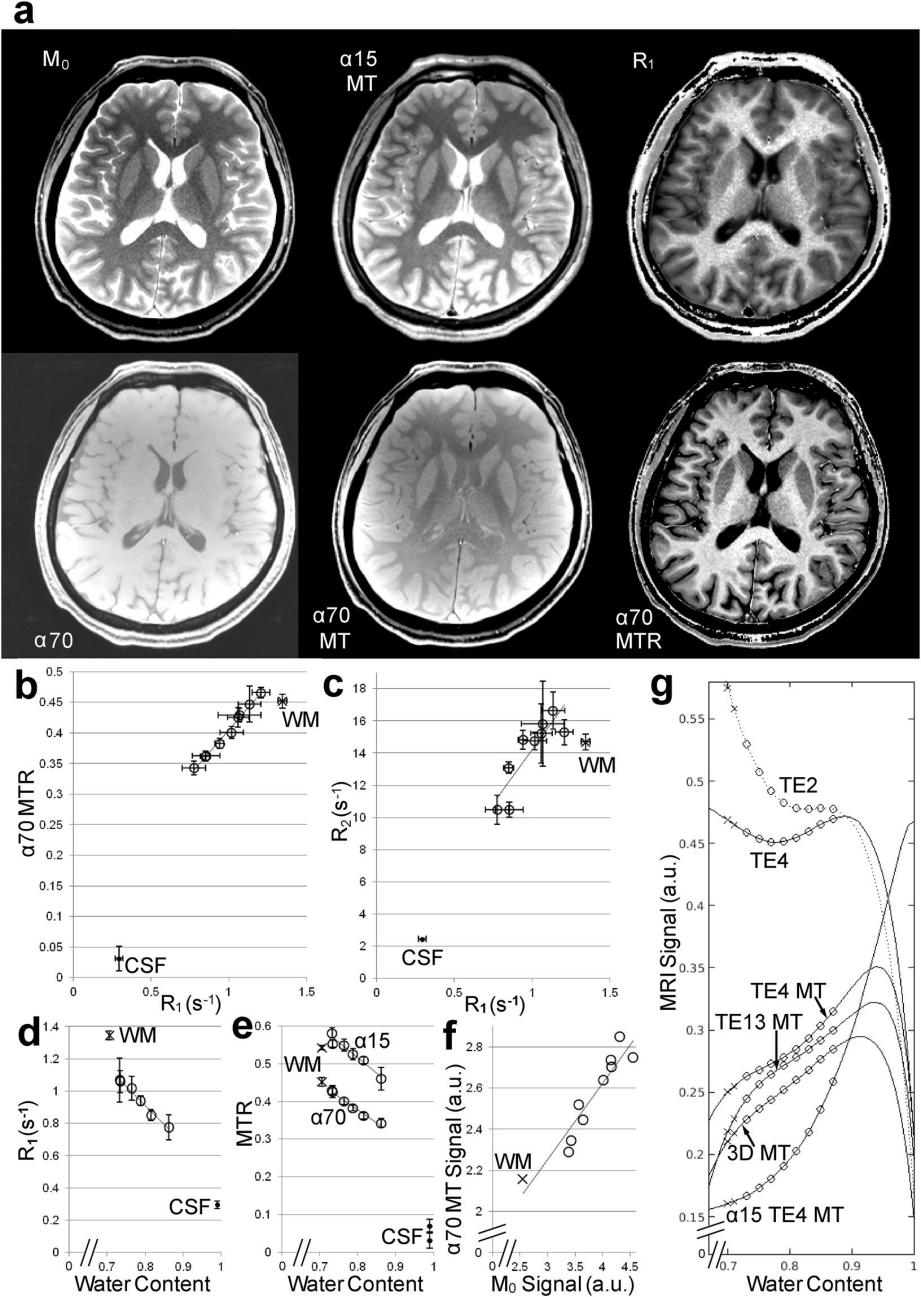
Magnetization-transfer MRI provides proton-density contrast in brain while saturating extracellular free water protons. **(a)** Comparison of M_0_ map (M_0_), proton-density-weighted MRI with MT (α15MT), R_1_ map (R_1_), T_1_-weighted MRI without (α70) and with MT (α70MT), MT ratio map (α70MTR). **(b)** Mean MT ratios (α70MTR) and **(c)** R_2_ of various regions in the brain plotted vs. their R_1_ as well as **(d)** R_1_ and **(e)** MT ratios (α15 MTR and α70 MTR) plotted vs. their water content. Functions and correlation coefficients are: (**b**) y = 0.2936x + 0.1116, r = 0.99b (**d**) y = −2.3556x + 2.796, r = −0.99, (**e**) y = −0.8181x + 1.1706, r = −0.97 for α15 and y = −0.6843x + 0.9263, r = −0.99 for α70. **(f)** Regional signal intensities in α70 MT plotted vs. those in M_0_ in a subject. They correlate significantly (r = 0.94, p < 0.0005). **(g)** Calculated signal intensities for RF-spoiled gradient-echo MRI as a function of the water content in human brain. (TE2) 2D, TR/TE = 250/2.46 ms, α 70°, (TE4 and TE4MT) 2D, TR/TE = 863/4.4 ms, α 70° without and with magnetization transfer, (TE13MT) 2D, TR/TE = 715/13.2 ms, α 70° with magnetization transfer, (3DMT) 3D, TR/TE = 47/7.5 ms, α 22°, (α15TE4MT) 2D, TR/TE = 863/4.4 ms, α 15° with magnetization transfer. Here, for a selected combination of acquisition parameters α, TR, and TE, the variables R_1_, R_2_*, and MTR used for the calculation of MRI signal can be described as a function of tissue water content as shown in Supplementary Fig. 3. The R_1_ and R_2_* are obtained from the functions given in the legend of Supplementary Fig. 3a and 3d, respectively. The MTRs for “TE4MT”, “TE13MT”, “3DMT”, and “α15TE4MT” are obtained from the functions given in the legend of Supplementary Fig. 3b, 3c, 3e, and 3f, respectively. ◦ = gray matter structure (prefrontal cortex, caudate nucleus, putamen, thalamus, globus pallidus, and substantia nigra for (**d**) and (**e**). In addition, subthalamic nucleus, red nucleus, and locus coeruleus for (**b**), (**c**), and (**f**), ×  = WM = frontal subcortical white matter, ∙ = CSF = cerebrospinal fluid, error bars = standard deviation.

Figure 2a and Supplementary Fig. 1 compare various quantitative maps of the human brain. The M_0_ map (M_0_), proton-density-weighted MRI with MT (α15 MT), and T_1_-weighted MRI with MT (α70 MT) reveal a similar contrast except for CSF. The MT ratio maps (α15 MTR and α70 MTR) and the R_1_ map (R_1_) also show similar contrast. Quantitative evaluations (Fig. 2b–f, Supplementary Table 2) confirm these observations. Mean regional MT ratios (α70 MTR) of gray matter (GM) structures correlate linearly with their longitudinal relaxation rate R_1_ (Fig. 2b). The deviation of the MT ratios of white matter (WM) and CSF from the linear relationship is attributable to their lower R_2_/R_1_ ratio (Fig. 2c) and thus to a weaker direct-saturation effect. Here, the WM shows a similar downward deviation because of its lower paramagnetic ion content^5^ (i.e., low R_2_) and its strong interaction with water protons^2^ (i.e., high R_1_). CSF, with its high water content, is also exposed to a lower direct-saturation effect because other molecules increase the R_2_ of the water protons more than R_1_. *In vitro* experiments of solutions with 6 different R_1_ and R_2_ values confirmed that direct-saturation effects correlate linearly with R_2_/R_1_ ratios (Supplementary Fig. 2a–c).

Mean regional R_1_ of GM structures correlate linearly with their water content (Fig. 2d). The upward deviation of the WM R_1_ from the linear relationship is again attributable to its large interaction with water protons^2^. This effective cross-relaxation in WM is apparently cancelled out by the more pronounced direct-saturation effect on GM structures, which results in an apparent linear relationship between the MT ratio (α70) and the water content including both GM and WM (Fig. 2e). Accordingly, the even more pronounced direct-saturation effect by a weaker on-resonance irradiation (α15) brings them again out of the apparent linear relationship. The values of the GM structures with significantly higher iron content, e.g., the globus pallidus and red nucleus, and those with significantly higher copper content, e.g., the substantia nigra (SN) and locus coeruleus (LC), are well in line with those of other GM structures. Paramagnetic ion concentrations correlate neither with R_1_ nor with MT ratios (Supplementary Fig. 2d–i). These findings indicate that the regional water content determines both MT ratio and R_1_. Accordingly, regional signal intensities in T_1_-weighted MRI with MT (α70 MT) correlate significantly (p < 0.005) with those in M_0_ in every subject (Fig. 2f), which also indicates that the regional spin density of intracellular water protons correlate significantly with the regional water content.

Figure 2g shows calculated signal intensities for gradient-echo MRI as a function of water content in human brain based on the obtained MT ratios as well as R_1_ and R_2_* values (Supplementary Fig. 3). A strong on-resonance irradiation with short TE and TR (TE2 in Fig. 2g) saturates the free water protons in the extracellular fluid with a water content^6^ of 0.99. This results in a typical T_1_-weighted MRI contrast and a high signal intensity for WM. A prolongation of echo time (TE4) reduces the T_1_-weighting and thus the WM signal. The addition of MT (TE4MT) suppresses the MRI signal in proportion to the macromolecular content (i.e., in inverse proportion to the water content), while the saturation of the free water protons is preserved. As a result, the MT effect dominates for brain tissue (water content < 0.9), while the T_1_ effect dominates for extracellular fluid (water content > 0.95). When saturating extracellular free water protons, the significant correlation between the signal intensities in MT-MRI and the proton density (Fig. 2f) indicates that the signal intensities reflect the spin density of intracellular water protons with short T_1_ values. This relationship between the MT-MRI signal intensity and the water content remains unaffected by a further prolongation of TE (TE13MT) or by a three-dimensional acquisition (3DMT). In contrast, a weak on-resonance irradiation in combination with MT (α15TE4 MT) leaves the extracellular free water protons unsaturated and provides a typical proton-density MRI contrast for both intra- and extra-cellular water (c.f., α15MT in Fig. 2a).

**Figure 3:**
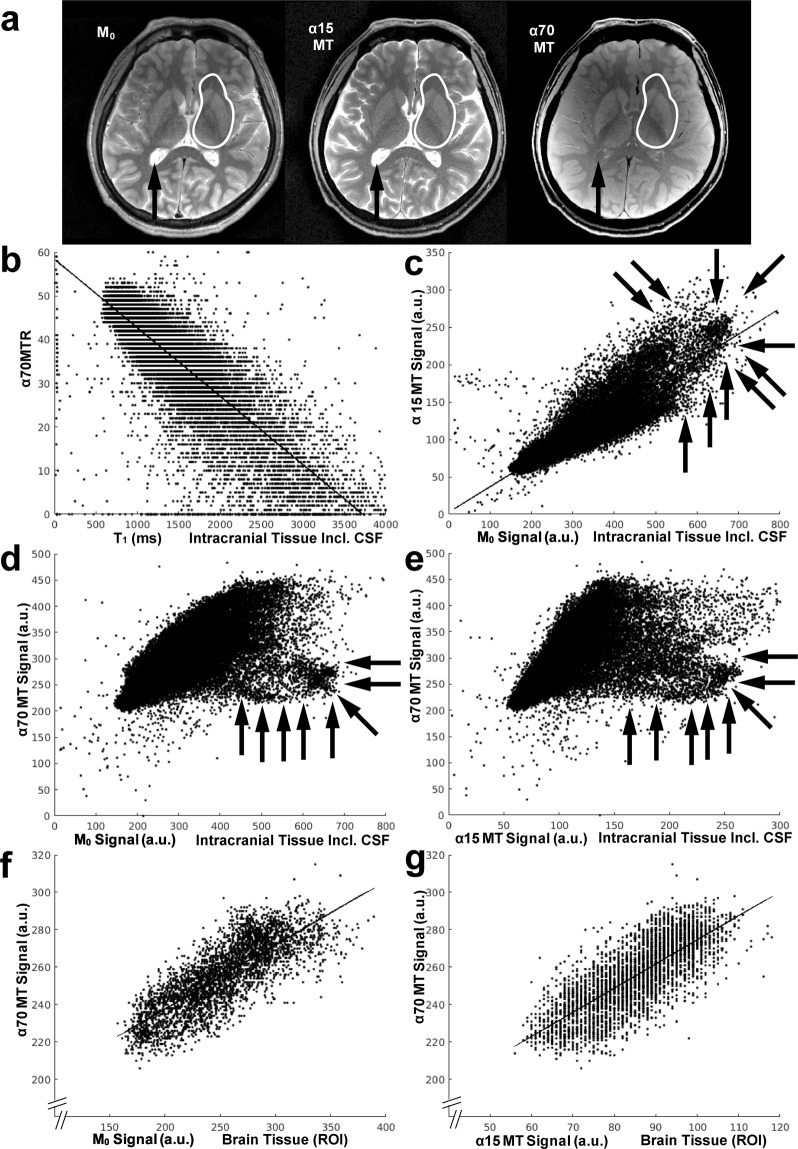
Magnetization-transfer MRI provides proton-density contrast in brain while saturating extracellular free water protons. (**a**) Comparison of M_0_ map and (α15MT) proton-density-weighted as well as (α70MT) T_1_-weighted MRI with magnetization transfer. Note the similar contrast between M_0_ and α15MT for the entire intracranial tissue, the similar contrast for all three images within the regions-of-interest selected in brain (white lines), as well as the saturation of free water protons in the cerebrospinal fluid (arrows) in α70MT. (**b**) Pixel-by-pixel plots of α70MTR vs. T_1_, (**c**) of signal intensities in α15MT vs. M_0_, (**d**) of signal intensities in α70MT vs. M_0_, (**e**) of signal intensities in α70MT vs. α15MT for the entire intracranial tissue, (**f**) of signal intensities in α70MT vs. M_0_, and (**g**) of signal intensities in α70MT vs. α15MT for the regions-of-interest shown in (**a**). Note the significant (p < 0.0005) correlation in (**b**), (**c**), (**f**), and (**g**). A pool of pixels (arrows) with high signal intensity in M_0_ as well as in α15MT can be identified in (**c**). Strong on-resonance irradiation (**d**,**e**) results in a downward deviation of a pool of comparable size. These pixels are supposed to be dominated by free water protons. Functions and correlation coefficients are: (**b**) y = −0.0157x + 58.4, r = −0.84, (**c**) y = 0.3396x + 2.86, r = 0.87, (**f**) y = 0.3423x + 169.2, r = 0.80, and (**g**) y = 1.2925x + 145.4, r = 0.77.

The highly significant correlations in pixel-by-pixel plots (Fig. 3) confirm that the water protons whose T_1_ is shortened by diamagnetic molecules are proportionally saturated by off-resonance irradiation (Fig. 3b) and that magnetization transfer generates proton-density contrast (Fig. 3c). A pool of pixels with high signal intensity in M_0_ as well as in α15MT can be identified in Fig. 3c. With stronger on-resonance irradiation (Fig. 3d,e), a pool of comparable size deviates from the otherwise clearly correlated plots. These pools are supposed to consist of pixels that are dominated by free water protons because the exclusion of CSF from the analysis (see regions-of-interest in Fig. 3a) results in a clear correlation again (Fig. 3f,g).

### Magnetization transfer delineates small cell assemblies in the central nervous system of human and mice *in vivo*

**Figure 4:**
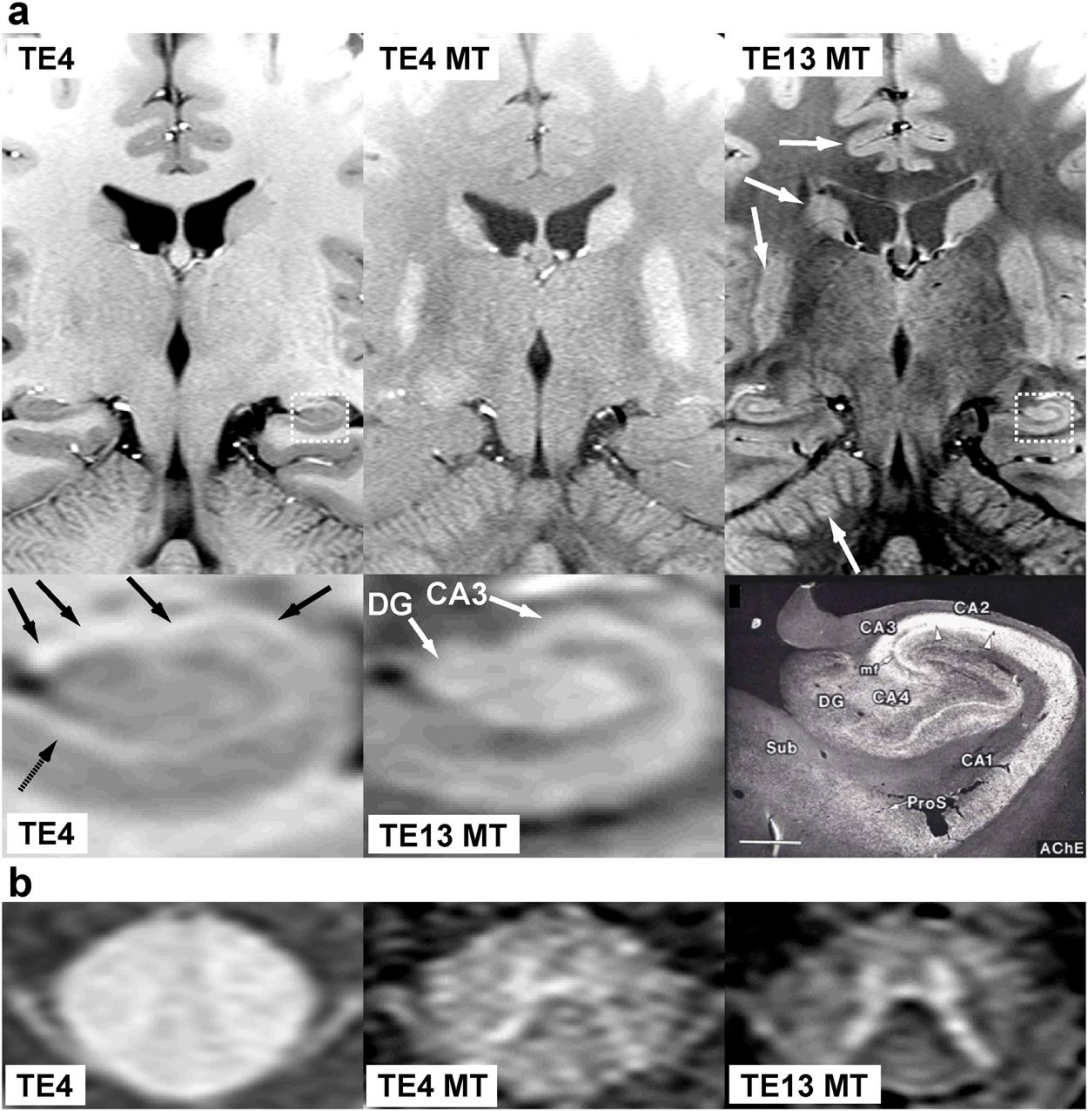
Magnetization transfer delineates cell assemblies in the central nervous system of human *in vivo*. **(a)** (Top row) (TE4) Coronal *in vivo* T_1_-weighted MRI (TR/TE = 473/4.4 ms, α 70) without magnetization transfer, (TE4 MT) with magnetization transfer, and (TE13 MT) with magnetization transfer and prolonged echo time (TR/TE = 715/13.2 ms). Arrows indicate gray matter structures, i.e., (from top to bottom) the cerebral cortex, caudate nucleus, putamen, and cerebellar cortex. (Bottom row) (TE4 and TE13 MT) Magnified MR images of the hippocampal formation *in vivo* indicated as dotted rectangles in top row images in comparison with (right) a light microscopic image of the acetylcholine esterase *ex vivo*^7^ (contrast inverted). Arrows indicate myelinated layers such as the alveus (black arrows) and the stratum lacunosum-moleculare (dotted arrows). In the light microscopic image, the cytoplasm and proximal dendrites of the neurons in the dentate gyrus as well as those in the stratum oriens, pyramidale, and radiatum of the CA1~CA4-prosubiculum are highlighted by the reaction products^7,8^. CA1~4 = Subfields 1~4 of the Ammon´s horn, DG = dentate gyrus, mf = mossy fibers, ProS = prosubiculum, Sub = subiculum. **(b)** (TE4) Axial MRI of the upper cervical spinal cord of a human subject without magnetization transfer, (TE4 MT) with magnetization transfer, and (TE13 MT) with magnetization transfer and prolonged echo time. Predominant saturation of the white matter by magnetization transfer preserves the bright H-shaped cell assemblies.

Figure 4 shows images obtained with the parameters given in Fig. 2g as “TE4”, “TE4 MT”, and “TE13 MT”. Repetitive on-resonance irradiation (TE4 in Fig. 4) saturates the CSF water protons while providing the brain tissue with high signal intensities. The magnified image of the hippocampal formation reveals high signal intensities for myelinated layers such as the alveus and the stratum lacunosum-moleculare. There is hardly a structure identifiable within the spinal cord. Magnetization transfer (TE4 MT in Fig. 4) predominantly suppresses the WM signal and shows the H-shaped cell assemblies as bright structures. A prolongation of TE (TE13 MT in Fig. 4) further enhances the image contrast and delineates the small cell assemblies in the hippocampal formation and the spinal cord even more clearly. The MRI pattern of high signal intensity in the hippocampal formation corresponds to the distribution pattern of the acetylcholinesterase reaction product (Fig. 4). This suggests that the MRI signals originate from cytoplasms and proximal dendrites of the neurons in the dentate gyrus as well as in the stratum oriens, pyramidale, and radiatum of the CA1~CA4~prosubiculum^7,8^. The pyramidal and polymorphic neurons that are rich in acetylcholine esterase play a critical role in establishing complicated neural networks^9^.

**Figure 5:**
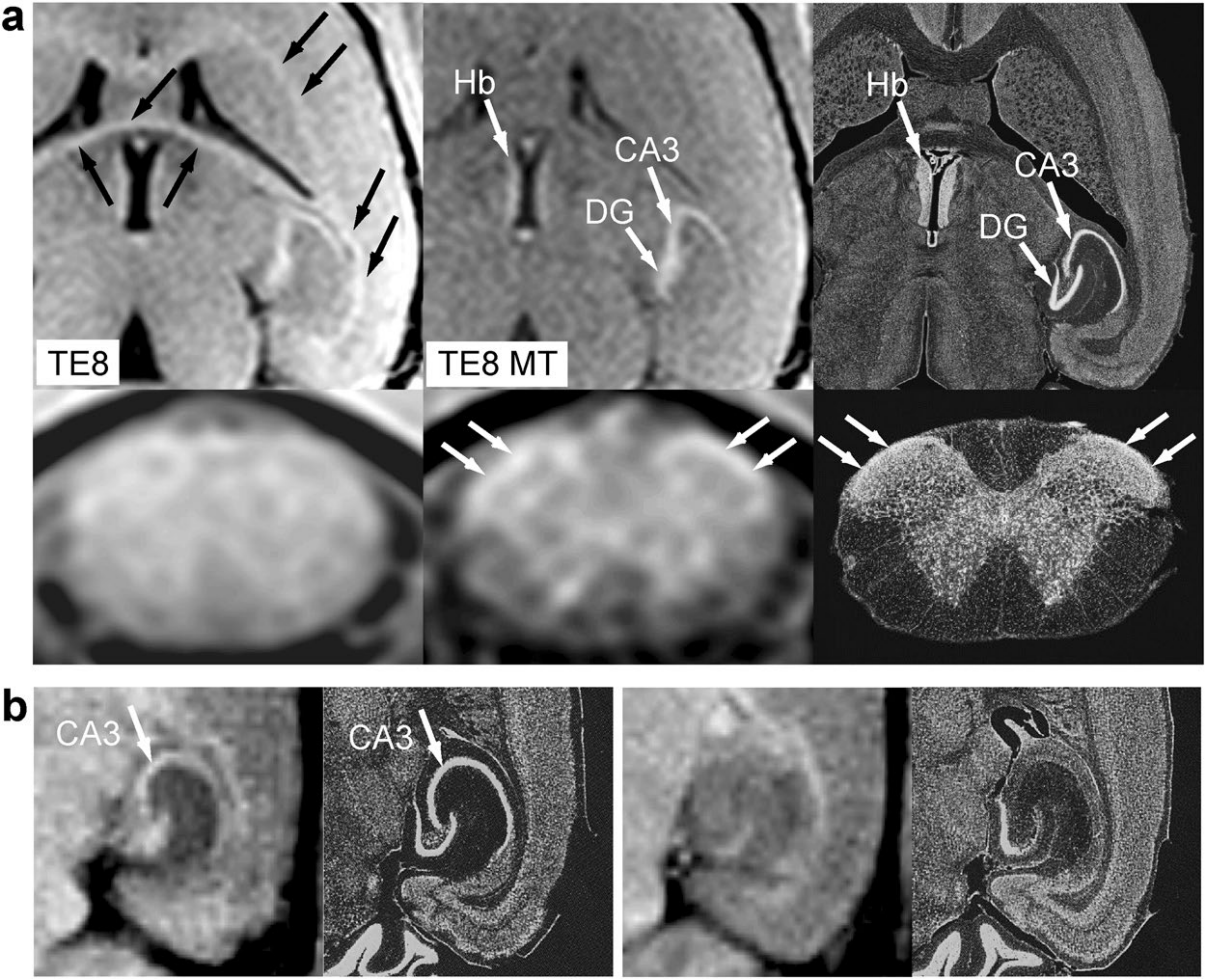
Magnetization transfer delineates cell assemblies in the central nervous system of mice *in vivo*. **(a)** (Top row) Horizontal sections of the forebrain as well as (bottom row) axial sections of the upper cervical spinal cord of mice obtained by (left column) *in vivo* T_1_-weighted MRI (2.35T, RF-spoiled 3D FLASH, TR/TE = 30/7.6 ms, α 25°, Δf = 5000 Hz, ω_SAT_ = 1045°/12 ms, 117 µm isotropic resolution, measuring time = 4 hours) without and (middle collumn) with magnetization transfer in comparison with (right column) light microscopy of cell bodies^49^ (contrast inverted). Magnetization transfer suppresses the signal intensities of the myelinated tissue and leaves the cell waters as bright structures. The white arrows indicate the unmyelinated cell layers of the Rexed lamina II of the spinal cord. Black arrows = myelinated tissue, Hb = habenular nucleus, DG and CA3 = dentate gyrus and CA3 of the hippocampal formation. **(b)** (Left) horizontal MRI *in vivo* (2.35T, RF-spoiled 3D FLASH, TR/TE = 22/8.2 ms, α 30°, 100 × 100 × 500 μm^3^ resolution) and corresponding histology (cresyl violet staining for cell bodies, contrast inverted) of the hippocampus of a placebo-treated FVB/N mouse and (right) a mouse 4 days after administration of kainic acid. The lack of pyramidal cells in the CA3 subfield of the lesioned animal corresponds to the lack of MRI contrast.

Similarly, in the brain of mice (Fig. 5a), magnetization transfer suppresses the signal intensities of the myelinated tissue and highlights cell assemblies as bright structures. In the spinal cord, MT distinguishes the unmyelinated cell layers of the Rexed lamina II (white arrows), the myelinated GM, and WM. These observations suggest unmyelinated layers that are tightly packed with nerve cell bodies which are responsible for the highest signal intensity. This assumption was verified by an additional experiment which compares a placebo-treated and a lesioned mouse (Fig. 5b). In agreement with previous observations of this well-established model for nerve cell death^10^, the histologic staining reveals a marked loss of pyramidal cell bodies in the affected animal. The corresponding loss of MRI signal intensity therefore supports the assignment of the high signal intensity to the presence of viable pyramidal cell bodies. As a consequence, the bright structure in T_1_-weighted MRI of the Ammon´s horn of the hippocampus with or without MT (Fig. 5a) must be ascribed to the densely packed layer of pyramidal cell bodies.

### Magnetization transfer delineates noradrenergic neuron groups in the brainstem of human and mice *in vivo*

**Figure 6:**
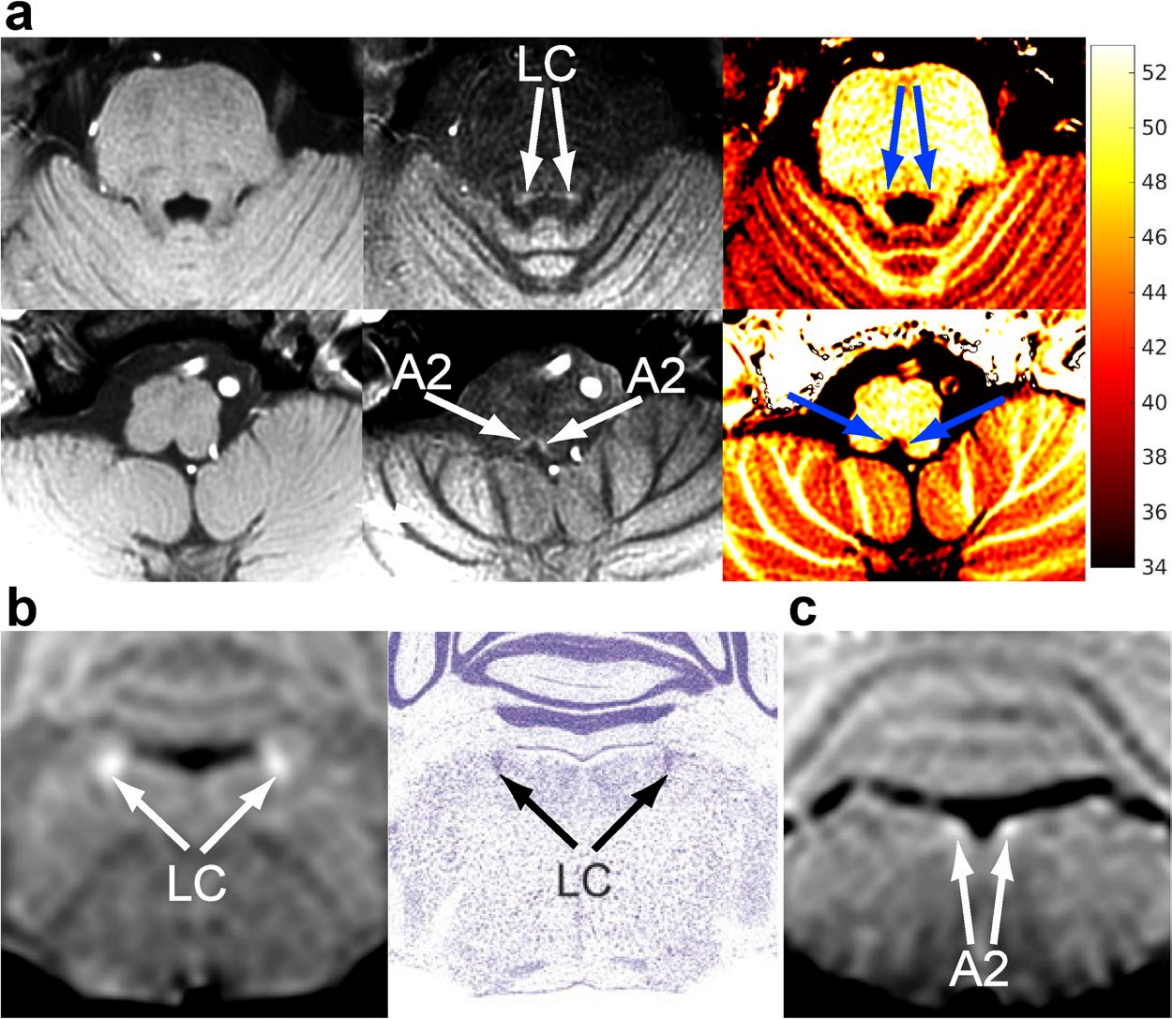
Magnetization transfer delineates noradrenergic neuron groups in the brainstem of human and mice *in vivo*. **(a)** (Top row) Transversal T_1_-weighted MRI (3T, spoiled 2D FLASH, TR/TE = 863/4.4 ms, α 70°, (0.66 mm)^2^ in-plane resolution, 2.5 mm slice thickness, 21 slices, total acquisition = 9 min 11 s) of the locus coeruleus and (bottom row) A2 cell groups of an adult human (left column) without magnetization transfer, (middle column) with magnetization transfer, and (right column) magnetization-transfer ratio map (scale: 34–53%). **(b)** (Left) Coronal MRI (spoiled 3D FLASH, TR/TE = 30/7.6 ms, flip angle 22°, Δf = 2500 Hz, ω_SAT_ = 523°/12 ms, 117 µm isotropic resolution) of the locus coeruleus of a 4-week-old female mouse in comparison with (right column) corresponding Nissl-stained sections (adapted from^50^). **(c)** Coronal MRI sections of the A2 cell groups of a 4-week-old female wild-type mouse.

In the brainstem, we delineated assemblies of noradrenergic neurons (Fig. 6a, Supplementary Fig. 4). Noradrenaline, also called norepinephrine, is a catecholamine that acts as hormone and neurotransmitter. Noradrenergic neurons, which produce the neuromodulator and release it from axonal terminals that spread widely throughout the brain, are assembled in the brainstem as A1 to A7 cell groups^11^. The major groups^12^ are A2, i.e., the dorsal motor vagus nucleus and nucleus tractus solitarius, in the medulla oblongata and A6, i.e., LC, in the pons. Here, we observed that the noradrenergic cell groups have low MT ratios and high T_1_ and T_2_ values compared to other GM structures (Supplementary Fig. 4, Supplementary Table 2). These findings indicate that the noradrenergic cell groups have high water content and that their high concentration of copper is not the source of the high MRI signal intensity.

**Figure 7:**
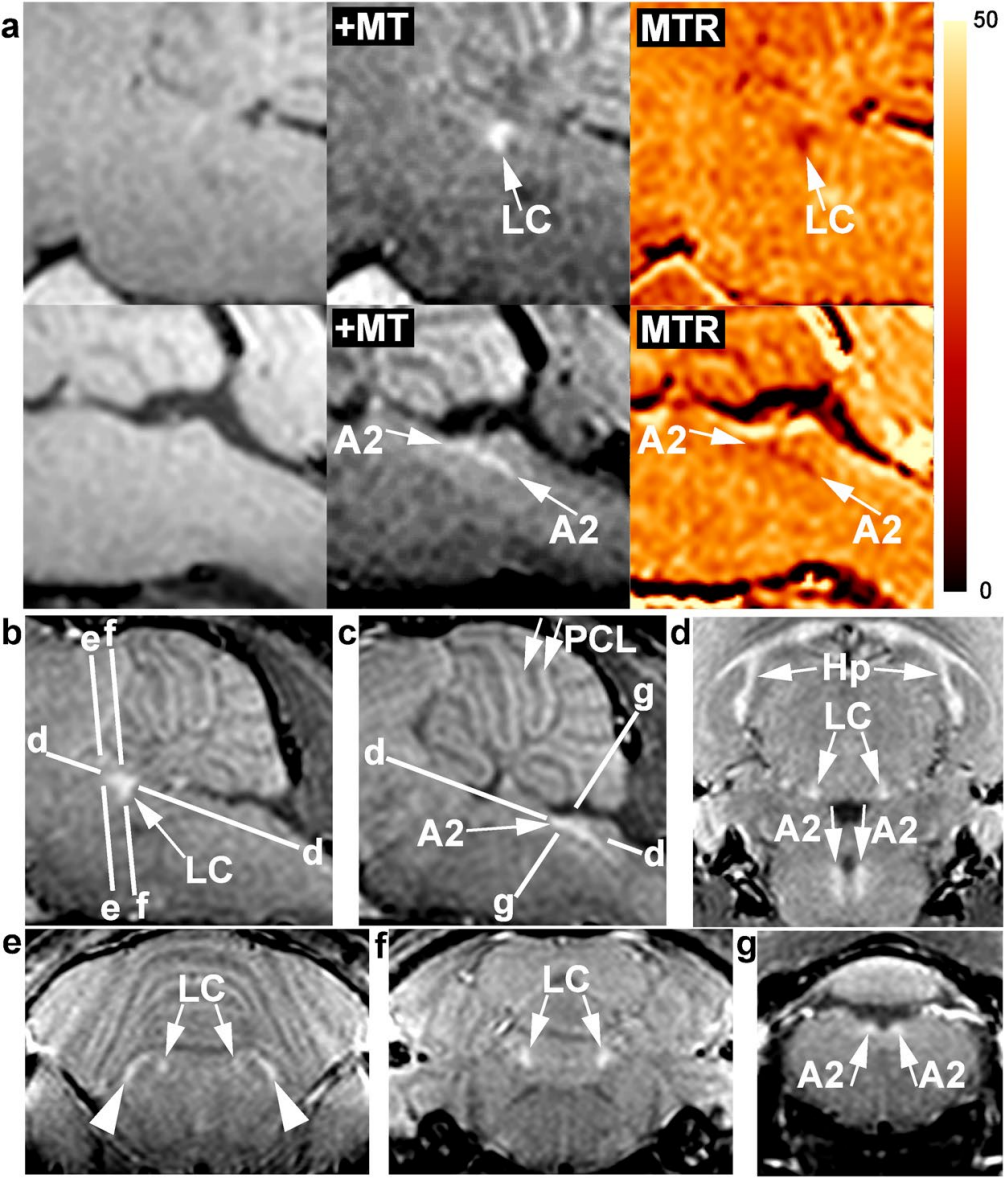
**(a)** (Upper row) Parasagittal T_1_-weighted MRI (2.35T, RF-spoiled 3D FLASH, TR/TE = 30/7.6 ms, α 25°, 117 µm isotropic resolution, total acquisition = 4 hours) of the locus coeruleus and (lower row) the A2 cell groups of an 8-week-old female NMRI mouse (left column) without magnetization transfer, (middle column) with magnetization transfer (Δf = 5000 Hz, ω_SAT_ = 1045°/12 ms), and (right column) magnetization-transfer ratio map (scale: 0–50%). **(b)** Parasagittal MRI (2.35T, RF-spoiled 3D FLASH, TR/TE = 30/7.6 ms, flip angle 22°, Δf = 2500 Hz, ω_SAT_ = 523°/12 ms, 117 µm isotropic resolution) of the locus coeruleus and **(c)** A2 cell group of a 3-week-old male NMRI mouse. **(d)** Horizontal and **(e**–**g)** coronal MRI of the same animal showing the planes indicated in (**b**,**c**). A2 = A2 cell group, Hp = pyramidal cell layer of the hippocampus, LC = locus coeruleus, PCL = Purkinje cell layer of the cerebellum, arrowheads = marginal nuclei of the brachium conjunctivum.

Similarly, with optimized parameters (Supplementary Tables 1a–h) we also delineated LC and A2 in mice (Fig. 6b,c, and Fig. 7). In T_1_ map, neither LC nor A2 group was delineated from surrounding brainstem. The T_1_ values (mean ± SD) of the brainstem at 2.35T are 0.93 ± 0.01 s at the level of LC and 0.95 ± 0.03 s at the level of A2 in males (n = 5), or 0.97 ± 0.02 s at the LC level and 1.0 ± 0.03 s at the A2 level in females (n = 4). The absence of T_1_ contrast and the T_1_ values are in line with data obtained in human brain at 3T (Supplementary Table 2). The lack of delineation does not result from the partial volume effect because T_1_-weighted MRI with MT at the same spatial resolution (117 × 117 × 234 µm^3^) delineates the LC and A2.

### Magnetization-transfer MRI detects signal enhancement of specific cell assemblies after administration of manganese

**Figure 8:**
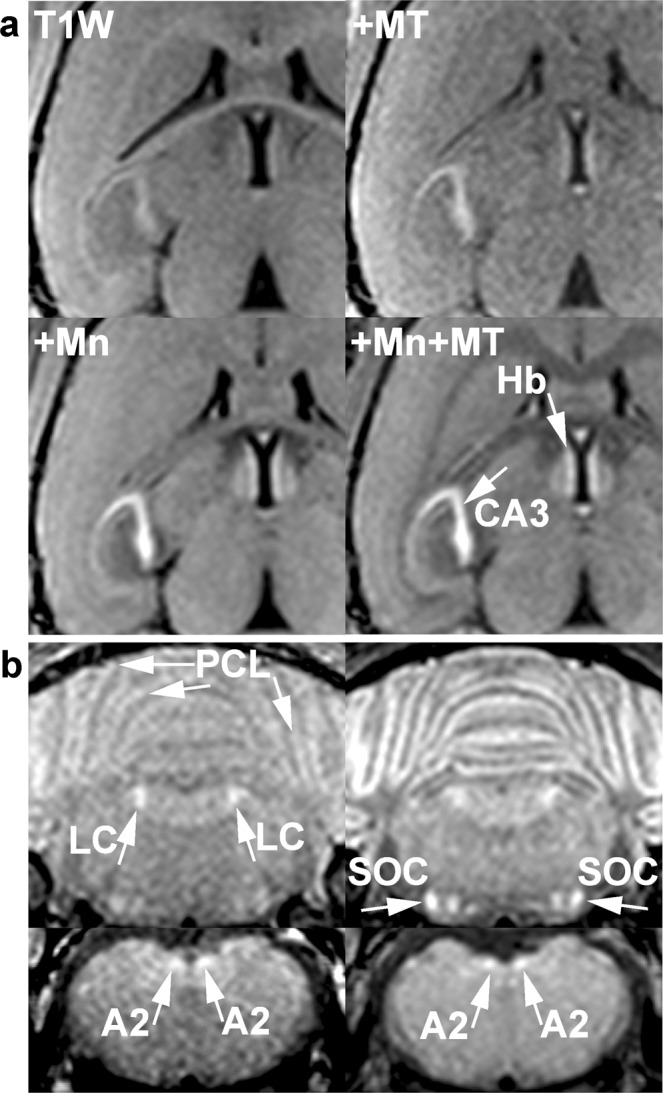
(**a)** (T1W) Horizontal T_1_-weighted images of the brain of a female young adult NMRI mouse, (+MT) with magnetization transfer, (+Mn) after Mn^2+^ injection, and (+MT + Mn) with MT after Mn^2+^ injection. Magnetization transfer delineates nerve cell assemblies that are further enhanced by the accumulation of intracellular Mn^2+^. **(b)** (Top row) Coronal MRI of the locus coeruleus and (bottom row) A2 cell groups of the same mouse (left) before and (right) after Mn^2+^ injection. The effect of Mn^2+^ on noradrenergic neurons is less pronounced than that on other nerve cell assemblies such as the cerebellar Purkinje cell layers or the superior olivary complexes. A2 = noradrenergic cell group 2, Hb = habenular nucleus, CA3 = CA3 of the hippocampal formation, LC = locus coeruleus, PCL = Purkinje cell layer of the cerebellum, SOC = superior olivary complex.

As mentioned earlier, physiological concentrations of endogenous paramagnetic ions have no significant effect on R_1_ or MT ratio (Supplementary Fig. 2d–i). On the other hand, as shown in Fig. 8 and Supplementary Table 3, we observed significant MT ratio reduction as well as significant signal increase in MT-MRI of the brain of mice after administration of Mn^2+^ in agreement with a number of previous studies^13^. The Mn^2+^-induced signal-to-noise ratio (SNR) increase for the pyramidal cell layers (81%, p < 0.01) and habenular nuclei (79%, p < 0.05) is significantly greater than for LC (49%). These findings indicate that the uptake and/or storage capacity of LC neurons for Mn^2+^ ions is limited and suggest that these mechanisms may be preferentially oriented towards Cu^2+^ ions.

## Discussion

### Generation of image contrast

In order to visualize cell assemblies by *in vivo* MRI, we exploited differences in spin density, T_1_, and T_2_* of water protons among different tissue compartments because these properties determine the observable magnetization in spoiled gradient-echo MRI. First of all, saturation of extracellular free water protons was accomplished by repetitive strong on-resonance irradiation which provides images where intracellular water protons exhibit high signals. Secondly, we used MT to exploit the spin-density difference between intracellular and lipid-associated water protons as the degree of MT saturation primarily depends on the relative concentrations of water and macromolecules^14^ (Fig. 2e). Thirdly, prolongation of TE further adds to the spin-density contrast because T_2_ and T_2_* are also largely a function of macromolecule concentration^15^ (Supplementary Fig. 3d). Potentially more pronounced off-resonance artifacts due to TE prolongation may be minimized by increasing the spatial resolution with only mild SNR reductions because the receiver bandwidth may be reduced accordingly. Taken together, we cancelled the T_1_ contrast in gradient-echo MRI with the use of MT and TE prolongation, which results in an image contrast that primarily reflects proton density (c.f., equations in the Methods and Fig. 2g) and only secondarily T_1_ or T_2_* relaxation. The proton-density effect dominates the observable signal for lipid-associated water protons (<0.9), while the T_1_ effect applies to the extracellular free water protons (>0.95).

In principle, signal intensity in gradient-echo MRI with strong on-resonance and off-resonance irradiation reflects the density of water protons whose T_1_ is shortened by paramagnetic ions. This is because water protons with exposure to diamagnetic molecules become proportionally saturated by off-resonance irradiation (Figs 2b and 3), while the free water protons remain saturated by on-resonance irradiation (Fig. 2g). In other words, the effect of water proton T_1_-shortening on MRI signal intensity (i.e., signal increase) induced by diamagnetic molecules is cancelled by the effect induced by off-resonance irradiation (i.e., signal reduction due to saturation transfer), whereas the effect of water proton T_1_-shortening on MRI signal intensity (i.e., signal increase) induced by paramagnetic ions is not. Accordingly, T_1_-shortening by paramagnetic ions increases the MT-MRI signal intensity. On the other hand, shortening of T_2_ or T_2_* by paramagnetic ions reduces the signal intensity not only as a result of the more pronounced dephasing, but also by the more pronounced direct-saturation effect^2^. Therefore, the relaxation enhancement factor^16^ of paramagnetic ions as well as their local concentration must be considered, because it has a great influence on T_1_ and T_2_ relaxation times of affected water protons as well as their ratio. Upon binding to macromolecules, the ions with a higher factor provide more efficient T_1_ shortening, more efficient T_1_/T_2_ ratio reduction, weaker direct-saturation effect, and thus more efficient signal enhancement in MT-MRI. With regard to the most concentrated paramagnetic ions in brain, the relaxation enhancement factor^16^ of Fe^3+^ on binding to a macromolecule is only 0.3 while that of Cu^2+^ is 10 and that of Mn^2+^ is 8. This is because Fe^3+^ ion has a much shorter electron relaxation time than Cu^2+^ or Mn^2+^ ion^17^. Given their high relaxation enhancement factors as well as their high intracellular concentration^5^ which meets their high metabolic demand, it is possible that Cu^2+^ and Mn^2+^ together play a role in high MT-MRI signal intensity of some specific cell assemblies, although we showed that the water proton density plays the central role (Fig. 2d–e, Supplementary Fig. 2). In fact, we demonstrated that administration of Mn^2+^ shortens T_1_ relaxation times, reduces MT ratio, and increases the signal intensity in MT-MRI^18^.

Within a cellular structure, water proton exchange is fast compared to T_1_ so that all water protons have the same T_1_. Moreover, the diffusion is efficient at mixing multiple pools within a cellular structure. However, in brain there are regions that restrict the diffusion of the water, that possess less cellular structure (i.e., the neuropil), or that represent CSF spaces. These contributions cause contrast in T_1_ maps of the brain (e.g., GM, WM, CSF spaces). If diffusion were efficient at mixing multiple pools of water protons throughout the brain, then it would eliminate all T_1_ differences. Instead, the observed T_1_ contrast indicates that there are brain regions where the diffusion is not efficient to mix multiple pools. In this regard, the present approach is advantageous because it readily provides higher spatial resolution compared to conventional T_1_ mapping and thus is expected to distinguish cellular structures in an unprecedented way. Nevertheless, we do not entirely exclude the contribution of extracellular water protons to the MRI signal by the present method.

In general, the direct-saturation effect alters the image contrast beyond the change caused by “pure MT” alone. This effect occurs during application of strong off-resonance irradiation, e.g., several kHz off the water resonance. In this scenario, samples with shorter T_2_ times lead to broader resonance linewidths, more direct radiofrequency saturation, and a larger MTR (Supplementary Fig. 2a–c). The fact that the slope of this line is smaller at higher flip angles is simply due to greater saturation by on-resonance irradiation and thus reduced residual magnetization for absorbing off-resonance irradiation.

### Paramagnetic ions in brain *in vivo*

Iron, the most concentrated transition metal in the brain, is not sufficiently concentrated to interfere with the interaction between the much more concentrated macromolecules and water protons. Even in the globus pallidus, the structure with the highest iron concentration, the MT ratios and T_1_ relaxation times are both within the range (Fig. 2b, Supplementary Fig. 2d,g) expected solely by the macromolecule and water content. In addition, Fe^3+^ has a short electron spin relaxation time. Thus, a potential binding interaction with macromolecules *in vivo* may further reduce its relaxivity^16^ by 70%. In fact, the T_1_ relaxivity of free Fe^3+^ of 7–13 L∙mmol^−1^∙ sec^−1^ is reduced^19^ to about 4 L∙mmol^−1^∙ sec^−1^ upon binding to ferritin. Brain iron, 90% of which is bound as Fe^3+^ to ferritin^20^, can thus be considered as poor T_1_-shortening ion acting primarily on T_2_ relaxation time^21^ because of its great magnetic moment (5.9). Accordingly, the globus pallidus and red nucleus as the structure with the highest iron content provide low MT-MRI signal intensity in proportion to their low proton density (Supplementary Fig. 1).

Copper, a transition metal with a capacity to gain and donate electrons, serves as a cofactor for electron transfer by important enzymes. In the brain of mammals, they include cellular respiration (cytochrome-c oxygenase), anti-oxidant defense (superoxide dismutase), and catecholamine synthesis/metabolism (tyrosine hydroxylase and dopamine β-hydroxylase). In addition, copper is involved in glutamate-dependent activity of hippocampal neurons^22,23^. Accordingly, copper is most concentrated in the noradrenergic^5,24^, dopaminergic, and glutamatergic neurons, which reflects their high metabolic demands for the metal^25^. Most copper ions in brain are bound to proteins and thus their distribution among different sub-cellular compartments is tightly regulated^24,25^. Therefore, the T_1_ relaxivity of Cu^2+^ in brain *in vivo* may be higher than that of Fe^3+^, because Cu^2+^ has a long electron spin relaxation time^17^. At 50 MHz, T_1_ relaxivity of free Cu^2+^ is^19^ about 0.5 L∙mmol^−1^∙ sec^−1^ that may be enhanced upon binding^16^ to be about 5 L∙mmol^−1^∙ sec^−1^ *in vivo*, whereas that of free Fe^3+^ is^19^ 6.9 L∙mmol^−1^∙ sec^−1^ that may be reduced^16^ to about 3 L∙mmol^−1^∙ sec^−1^. T_2_ relaxivity of Cu^2+^ is much lower than Fe^3+^ because of its small magnetic moment^19,26^ (1.7–2.2). Given (i) these favourable properties for Cu^2+^ to efficiently reduce T_1_ and T_1_/T_2_ ratio as well as (ii) the high MT-MRI signal intensities of LC, A2, SN, and hippocampal neurons that may correspond to the high metabolic demand for copper^25^ in their noradrenergic, dopaminergic, or glutamatergic neurons, it is possible that intracellular copper concentration plays a role in MT-MRI signal intensity, although we showed that it is their high proton density that plays the central role.

Manganese is less concentrated in brain than iron or copper as long as its uptake into the systemic blood circulation is physiologically regulated by the hepatic portal system. Thus, the physiological brain concentration of manganese has no effect on MT ratios or T_1_ (Supplementary Fig. 2f,i). After administration of exogenous Mn^2+^ ions, however, the T_1_ relaxation times, MT ratio, and MRI signal intensity in brain alter significantly^18^. After entering the systemic blood circulation and crossing the capillary endothelium, Mn^2+^ ions are supposed to accumulate in the cytosol, mitochondria, lysosomes, and microsomes of brain cells, where they may bind to cytosolic proteins and inner mitochondrial membranes^13^. The resulting immobilization of Mn^2+^ ions may increase their T_1_ relaxivity^16^ *in vivo*, which reduces the T_1_/T_2_ ratio efficiently.

It is unlikely that the MT contrast of specific cellular assemblies shown in the present study is due to unique lipid or protein structures. If the high signal intensity of those structures were due to the presence of T_1_-shortening molecules, then the T_1_ of those structures would have been shorter than other gray matter or surrounding structures. The present study showed that exactly the opposite is the case (Supplementary Table 2). Given the long T_1_ and low MTR for those structures compared to other GM, the high MRI signal must be attributed to abundant water protons interacting with any T_1_-shortening paramagnetic ions rather than to abundant T_1_-shortening molecules. It is not the T_1_-relaxation but the proton density that determines the *in vivo* MT-MRI contrast within the brain (Figs 2, 3). Theoretically (c.f., equations in Methods section), T_1_-shortening plays a role, but the MRI signal intensity is dominated by M_0_. In this regard, low MT ratios and high T_1_ and T_2_ values of SN, LC, and A2 as well as of caudate nucleus and putamen (Supplementary Table 2) indicate that these structures have high water content and thus yield high signal intensities in MT-MRI (Supplementary Figs 1, 4). Conversely, high MT ratios and low T_1_ and T_2_ values of thalamus, globus pallidus, red nucleus, and subthalamic nucleus (Supplementary Table 2) indicate that these structures have low water content and thus yield low signal intensities in MT-MRI (Supplementary Fig. 1).

More specifically, the similar MRI contrast described for SN and LC in human brain so far has been attributed to a T_1_-shortening of intracellular water protons induced by neuromelanin^27,28^. Here we showed that cell assemblies like caudate nucleus and putamen that contain no neuromelanin yield as high intensities as SN and LC. Further, we demonstrated high MRI signal intensities in LC and A2 of 3-week-old mice as well (Fig. 7b-g). Given the lack of neuromelanin in rodents^29,30^, the high intensities of the catecholaminergic neurons are not attributable to neuromelanin but reflect their high water content. Even when assuming that paramagnetic ions contributed to their high signal intensities, the neuromelanin is unlikely to play a major role. The concentration of neuromelanin Fe in LC (SN) is only about 3.6 (20) ng/mg wet tissue, whereas the concentration of Fe in LC (SN) is as much as about 25 (150) ng/mg wet tissue^31^. The concentration of neuromelanin Cu in LC (SN) is only about 1.2 (0.4) ng/mg wet tissue, whereas the concentration of Cu in LC (SN) is as much as 31 (16) ng/mg wet tissue.

## Conclusions

To summarize, this is the first report about the *in vivo* MRI visibility of hippocampal and A2 cell assemblies in human as well as of noradrenergic neurons in animals. The generation of image contrast relies upon a simultaneous saturation of extracellular free water protons and lipid-associated water protons, preserving the signals from the water protons that are associated with intracellular paramagnetic ions. The image signal intensity is thus largely attributable to the density of the paramagnetic-ion-associated intracellular water protons and is shown to be unaffected by physiological concentrations of endogenous paramagnetic ions. Only when exogenous Mn^2+^ ions with their high relaxation enhancement factor are delivered to the brain in high concentration, the signal intensity showed its T_1_-dependence. Given that the decline of LC neuron numbers and the resulting noradrenalin shortage in brain are associated with aging, dementia, A*β* plaque load, and the progression of Alzheimer’s disease^32^, it is foreseeable that MRI of noradrenergic neuron groups as well as of the hippocampal cell assemblies in both laboratory animals and humans will play an increasing role in translational biomedical research of neurodegenerative diseases and beyond.

## Methods

### MRI of human brain and spinal cord

At 3T (Magnetom Prisma, Siemens Healthcare, Erlangen, Germany) transversal MRI (2D FLASH, TR/TE = 863/4.4 ms, pixel bandwidth = 140 Hz, (0.66 mm)^2^ resolution, 2.5 mm slice thickness, 21 slices, total acquisition = 4 min 35 s) was performed with the use of a 64-channel head coil. An on-resonance flip angle α of 70° was used for T_1_-weighted MRI while α of 15° was used for proton-density-weighted MRI. For off-resonance irradiation, magnetization transfer (10 ms Gaussian pulse, frequency offset = 1200 Hz, flip angle = 208.5°, amplitude = 27.9 V) and fat saturation were applied at every interleaved slice (every 41 ms) as provided by the manufacturer. T_1_ mapping^33,34^ as well as T_2_ and M_0_ mapping (Model-based Accelerated T2 Mapping, Siemens) were performed at the same spatial resolution. Regions-of-interests are selected in the frontal subcortical white matter, prefrontal cortex, caudate nucleus, putamen, thalamus, globus pallidus, subthalamic nucleus, red nucleus, and substantia nigra. Regional values are compared to each other and to the mean regional R_2_* values^35–37^ as well as to the mean regional content of water^38,39^, iron^40^, copper^41^, and manganese^42^.

For magnitization-transfer MRI of LC and A2, the data were accumulated twice (TR/TE = 863/4.4 ms, total acquisition = 9 min 10 s). The slices are positioned perpendicular to the anterior wall of the fourth ventricle. The LC is observable in the 5–7th slices from the top, while A2 is observable in the 16–18th.

For high-resolution magnetization-transfer MRI of the hippocampal formation and the upper cervical spinal cord, T_1_-weighted MRI (2D FLASH, α 70°, 0.38 × 0.76 mm^2^ in-plane resolution, 2.5 mm slice thickness, 11 slices) was performed with moderate echo time (TR/TE = 473/4.4 ms, pixel bandwidth = 140 Hz, two averages, total acquisition = 8 min 49 s) or with prolonged echo time (TR/TE = 715/13.2 ms, pixel bandwidth = 30 Hz, total acquisition = 8 min). For off-resonance irradiation, magnetization transfer (10 ms Gaussian pulse, frequency offset = 1200 Hz, flip angle = 208.5°, amplitude = 27.9 V) and fat saturation were applied at every interleaved slice (every 43 ms and 65 ms, respectively) as provided by the manufacturer.

### Calculation of signal intensity in gradient-echo MRI of the brain

In MRI of the brain *in vivo*, the signal originates exclusively from water protons. With signal contribution from T_2_ coherence being negligible, the observable magnetization in the steady state of spoiled gradient-echo MRI yields:

$${{\rm{S}}}_{0}={{\rm{M}}}_{0}\frac{1-{{\rm{e}}}^{-{\rm{TR}}\times {{\rm{R}}}_{1}}}{1-\,\cos \,{{\rm{\alpha }}e}^{-{\rm{TR}}\times {{\rm{R}}}_{1}}}{{\rm{e}}}^{-{\rm{TE}}\times {{\rm{R}}}_{2}^{\ast }}$$

with flip angle α, repetition time TR, echo time TE, spin-lattice relaxation rate R_1_, effective spin-spin relaxation rate R_2_*, and initial magnetization M_0_, while the observable magnetization in magnetization-transfer MRI yields

$${\rm{S}}={{\rm{S}}}_{0}(1-{\rm{MTR}})$$

with the observable magnetization in gradient-echo MRI without magnetization transfer S_o_ and magnetization-transfer ratio MTR.

### Animals and anesthesia

Mice were housed in groups under standard conditions at a temperature of 22 °C and a 12 h light/dark cycle with *ad libitum* access to standard food and water. A total of 32 mice were used. Firstly, 24 mice (NMRI, 6 male and 18 female, 3–8 weeks old) were used for the optimization of magnetization transfer. Secondly, to elucidate the nature of MRI contrast in the hippocampus, FVB/N mice (*n* = 2, male, 9 weeks old) were treated with a kainic acid lesion model^43^. FVB/N mice received a single subcutaneous injection of kainic acid (30 mg/kg, pH 7.3) or placebo (saline). During a subsequent 3 h observation period for behavioral symptoms, the lesioned animal showed signs of epileptic activity, including staring, decreased motility, twitching, rearing, and falling. MRI was performed before administration of kainic acid or placebo as well as 4 days later. Immediately after the second MRI examination, animals were transcardially perfused with neutral phosphate-buffered formalin (10%). Horizontal brain sections were cut at a thickness of 40 μm. Every third section was stained with cresyl violet to determine nerve cell loss. Thirdly, six mice (NMRI, female, 8–12 weeks old) were used for the manganese uptake study.

After induction of anesthesia with 5% isoflurane, animals were intubated with a purpose-built polyethylene endotracheal tube (0.58 mm inner diameter, 0.96 mm outer diameter) and artificially ventilated using an animal respirator (TSE, Bad Homberg, Germany) with a respiratory rate of 25 breaths per minute and an estimated tidal volume of 0.35 ml as previously described^44–46^. The animals were then placed in a prone position on a purpose-built palate holder equipped with an adjustable nose cone. The Göttingen animal bed^47^ secured a reproducible and reliable fixation of the mouse head and receiver coil in the magnet isocenter. Respiratory movement of the abdomen as well as rectal temperature was monitored by a unit supplied by the manufacturer (Bruker Biospin MRI GmbH, Ettlingen, Germany).

### Optimization of magnetization transfer in mouse brain

At 2.35T, MRI measurements were carried out using a 4.7/400 mm magnet (Magnex Scientific, Abingdon, UK) equipped with 200 mT m^−1^ gradients (Bruker Biospin MRI GmbH, Ettlingen, Germany). RF excitation and signal reception was accomplished with the use of a Helmholtz coil (inner diameter 100 mm) and an elliptical surface coil (inner diameter 20 mm × 14 mm), respectively. For the optimization of MT MRI, an off-resonance Gaussian RF pulse with a duration of 12 ms, a frequency offset of 2200–5000 Hz, and a mean amplitude of 50–200 Hz (flip angle 261°–1045°) was incorporated into a gradient-echo MRI sequence (RF-spoiled 3D FLASH, TR/TE = 30/7.6 ms, flip angle = 10°–30°, field-of-view = 30 × 18.75 × 22.5 mm^3^, matrix = 256 × 160 × 192 interpolated to 512 × 512 × 512, 8 averages, measuring time = 123 minutes) at 117 µm isotropic resolution. MTR was obtained from acquisitions with and without off-resonance irradiation.

We started with the use of 12 ms off-resonance pulse with mean amplitude of 200 Hz and a frequency offset of 5000 Hz, because this was found to be optimal for magnetization transfer in mouse brain *in vivo*^48^. With the use of this off-resonance pulse, we first tried to find out an optimal flip angle for the on-resonance pulse in 3D gradient echo MRI in order to delineate the LC and A2. As shown in Supplementary Table 1a, a flip angle of 22° turned out to be optimal. A flip angle of 18°~22° turned out also to be optimal for 12 ms off-resonance pulse with a mean amplitude of 100 Hz and a frequency offset of 2500 Hz (Supplementary Table 1b). Next, we found out that for the off-resonance pulse with a mean amplitude of 200 Hz, a frequency offset turned out to play no major role within the range between 3000 and 7000 Hz (Supplementary Table 1c). For amplitude of 100 Hz (Supplementary Table 1d), 2500–2800 Hz offset turned out to be optimal. For each respective optimal frequency offsets, the use of a lower amplitude resulted in a lower contrast-to-noise ratio (Supplementary Table 1e,f). The comparison of these two different amplitudes with respective optimal offsets (Supplementary Table 1g) shows that there was no substantial difference between them as far as contrast-to-noise ratio is concerned, because the greater amplitude generally resulted in lower signal-to-noise ratio. The use of the off-resonance pulse with a mean amplitude of 200 Hz and a frequency offset of 5000 Hz yielded the mean signal-to-noise ratio of LC to be 33.9–36.0 and of A2 to be 35.2–36.4, while with a mean amplitude of 100 Hz and a frequency offset of 2500 Hz yielded the mean signal-to-noise ratio of LC to be 36.7–39.2 and of A2 to be 37.8–39.6 (Supplementary Table 1a–g). There was also no difference between males and females (Supplementary Table 1h).

### T_1_ mapping of mouse brain

T_1_ relaxation times of LC and A2 were determined using a spin-echo multiple TR saturation recovery method (TE 16 ms, field of view 30 mm × 30 mm, matrix 256 × 256, in-plane resolution 117 µm × 117 µm, slice thickness 234 µm, five slices). TR was varied through 200, 400, 800, 1500, 3000, 5000 ms, six averages, and the total measuring time 209 min. Coronal MRI slices were selected to include LC and A2. The mean MRI signal intensity of each pixel was fitted for each TR value with the standard single exponential function to yield T_1_ values.

### Manganese uptake study

Each mouse received manganese chloride (0.12 mmol/kg body weight) via subcutaneous injection. The mice were returned to a chamber with unlimited access to food and water. Before and 3 days after Mn^2+^ injection, MRI measurements were carried out at 2.35T. An off-resonance RF irradiation with a frequency offset of 5 kHz and a mean amplitude of 200 Hz (flip angle 1045°) was incorporated into a T_1_-weighted gradient-echo MRI sequence (RF-spoiled 3D FLASH, TR/TE 30/7.6 ms, α 25°) at 117 µm isotropic resolution. For evaluation of signal intensities, regions-of-interest were selected in LC, other nerve cell assemblies (hippocampal formation, habenula, cerebellar cortex), cerebral cortex, and in the white matter (corpus callosum, cerebellar white matter).

### Mouse brain MRI evaluation

For evaluation of signal intensities, anatomically defined cross-sections were obtained from the original 3D MRI data sets by multiplanar reconstructions using software supplied by the manufacturer (Paravision 5.0, Bruker Biospin MRI GmbH, Ettlingen, Germany). The plane of the anterior commissure – posterior commissure served as a reference for the selection of standardized sections to facilitate comparisons with minimized intra- and inter-individual variability. For LC or A2, a rectangular region-of-interest of 6 pixels was taken in the center of the structures. For the brainstem, a circular region-of-interest of 1004 pixels was taken in the brainstem between LC and A2. SNR was defined as the mean MRI signal intensity divided by the standard deviation of the noise. The contrast-to-noise ratio was obtained by taking the difference between the SNR values. The analysis followed a strategy previously developed for intra-individual comparisons of MR images obtained after manganese administration^45^. Statistical evaluation was performed using SPSS^®^ (version 21.0, IBM^®^) and Microsoft Excel software. Significant differences between two groups of data were determined by the Mann-Whitney’s U-test.

### Compliance with Ethical Standards

All procedures involving human participants in this study were approved by the institutional committee of the Georg-August-Universität Göttingen and performed in accordance with the ethical standards of the institutional and national research committee and with the 1964 Helsinki declaration and its later amendments. All participants gave written informed consent before each examination.

All animal experiments were performed in accordance with German animal protection laws after approval by the responsible governmental authority (Landesamt für Verbraucherschutz und Ernährungssicherheit, Braunschweig, Germany) as well as by the institutional review board for animal protection.

### Third party rights

Images and drawings were taken or created by an author of the paper.

## Supplementary information

Supplementary Info

## Data Availability

The datasets generated during and/or analysed during the current study are available from the corresponding author on reasonable request.
