# Supplementary material for: Magnetic resonance imaging of brain cell water
Source: Sci Rep. 2019 Mar 25;9:5084. doi: 10.1038/s41598-019-41587-2 (PMC6434048; doi:10.1038/s41598-019-41587-2)
Supplement: Supplementary file 1 — Supplementary Info [file 41598_2019_41587_MOESM1_ESM.docx]

**Magnetic resonance imaging of brain cell water**

Takashi Watanabe*, Xiaoqing Wang, Zhengguo Tan, Jens Frahm

Biomedizinische NMR, Max-Planck-Institut für biophysikalische Chemie, Göttingen, Germany


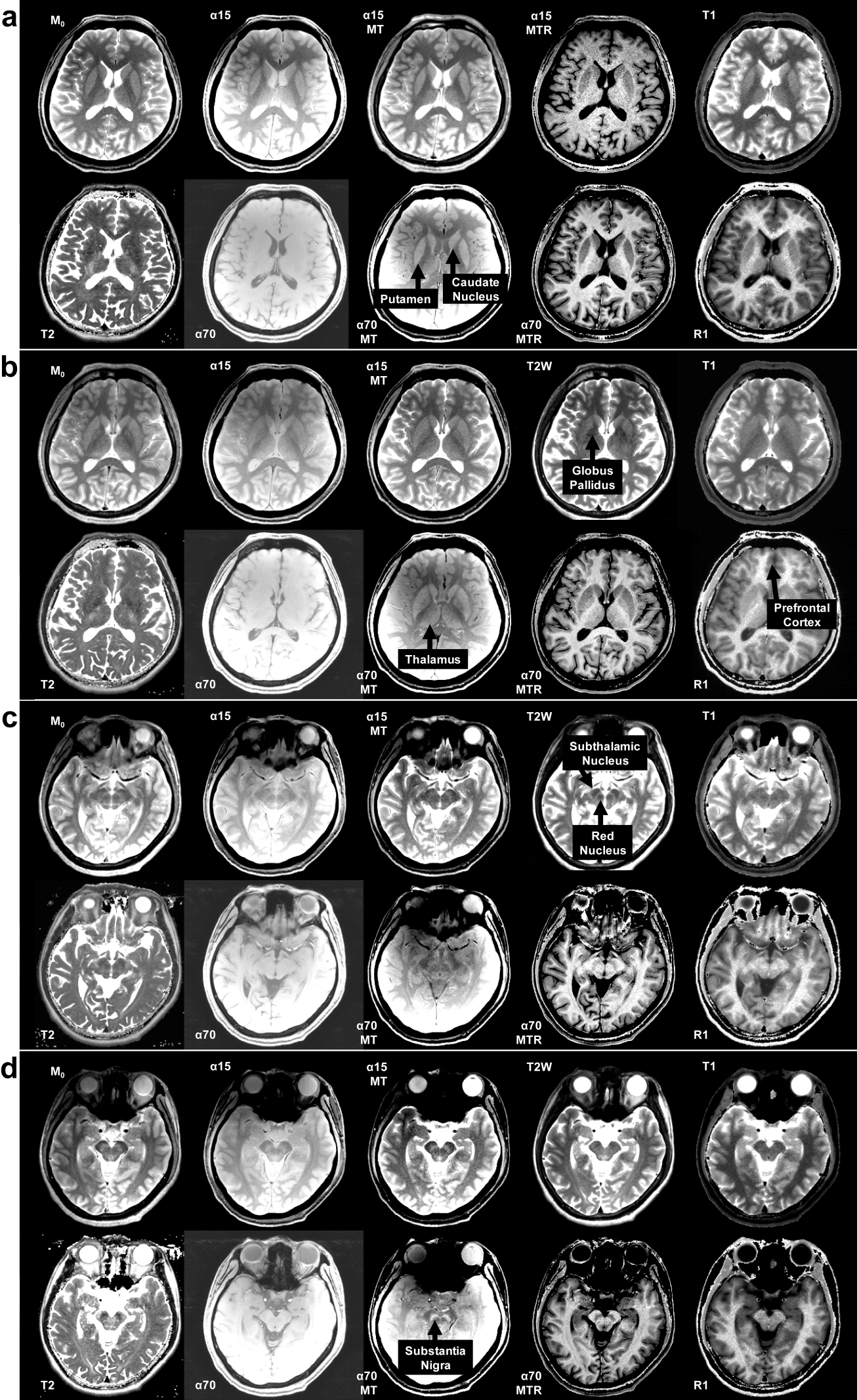
 **Supplementary Figure 1** (a) Transversal M_0_ map (M_0_), proton-density-weighted MRI without (α15) and with MT (α15 MT), MT ratio map (α15 MTR), T_1_ map (T1), T_2_ map (T2), T_1_-weighted MRI without (α70) and with MT (α70 MT), MT ratio map (α70 MTR), and R_1_ map (R1) showing the caudate nucleus and putamen, (b) globus pallidus, thalamus, and prefrontal cortex, (c) subthalamic and red nuclei, and (d) substantia nigra.


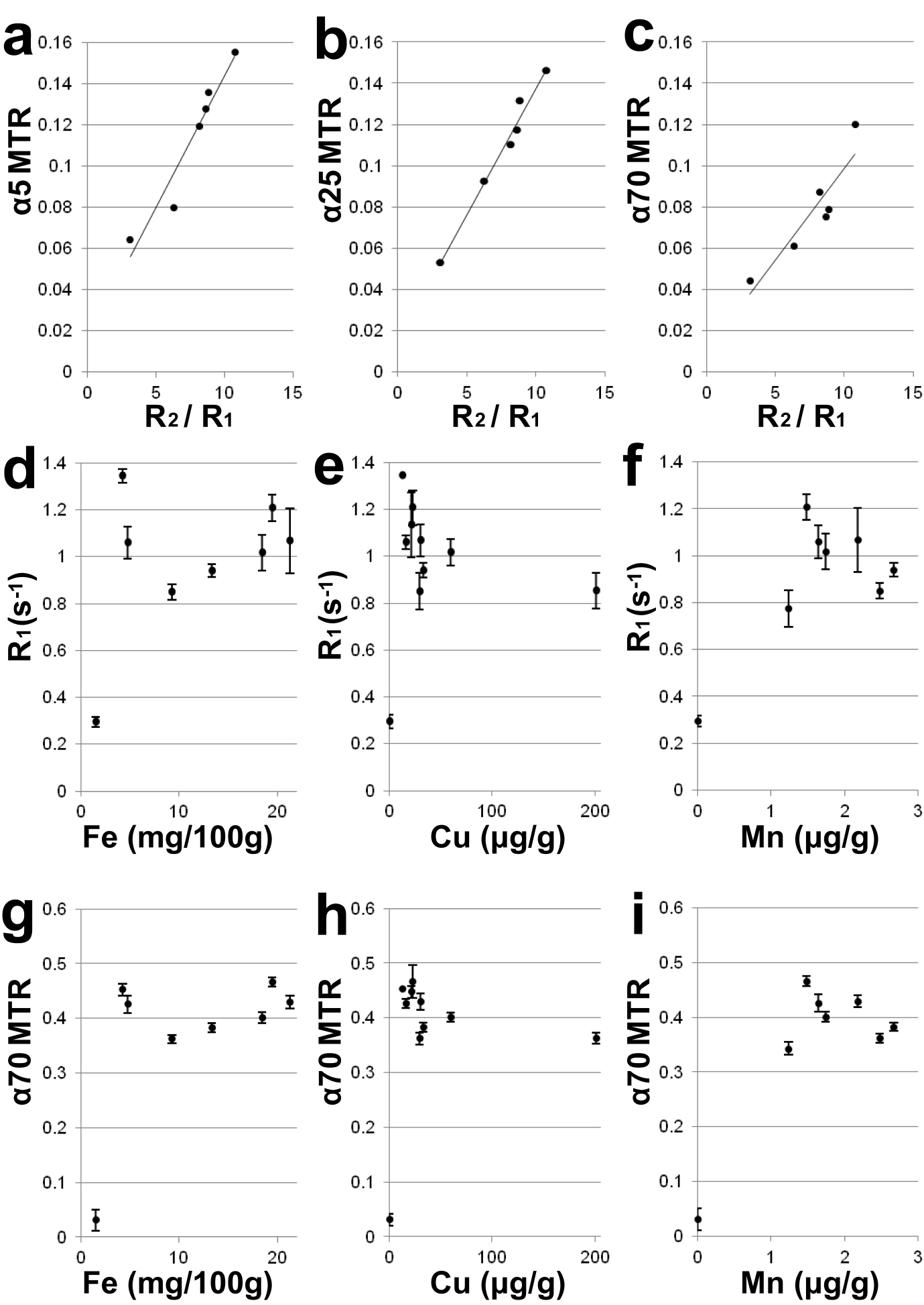


**Supplementary Figure 2** (a) Magnetization-transfer ratio of six different solutions *in vitro* (Test Object TO5, Tube numbers 3, 4, 7, 10, 14, and 16, Diagnostic Sonar Ltd, Livingston, Scotland) obtained by 2D FLASH (TR/TE = 1280/4.4 ms) with on-resonance flip angle of 5°, (b) 25°, and (c) 70° plotted vs. the R_2_/R_1_ ratio of each solution. Note the linear relationships between the direct-saturation effect and the R_2_/R_1_ ratio as well as the weaker direct-saturation effects by higher flip angles. Equations and correlation coefficients are: (a) y = 0.0128x + 0.0159, r > 0.99, (b) y = 0.0123x + 0.0142, r = 0.99, (c) y = 0.0089x + 0.0097, r = 0.92. (d) R_1_ plotted vs. the non-haemin iron content (mg iron / 100 g fresh weight), (e) vs. the copper content (μg/g dry weight), and (f) vs. the manganese content (μg/g dry weight) as well as (g) magnetization-transfer ratio (2D FLASH, TR/TE = 863/4.4 ms, α = 70°) plotted vs. the non-haemin iron content, (h) copper content, and (i) manganese content.


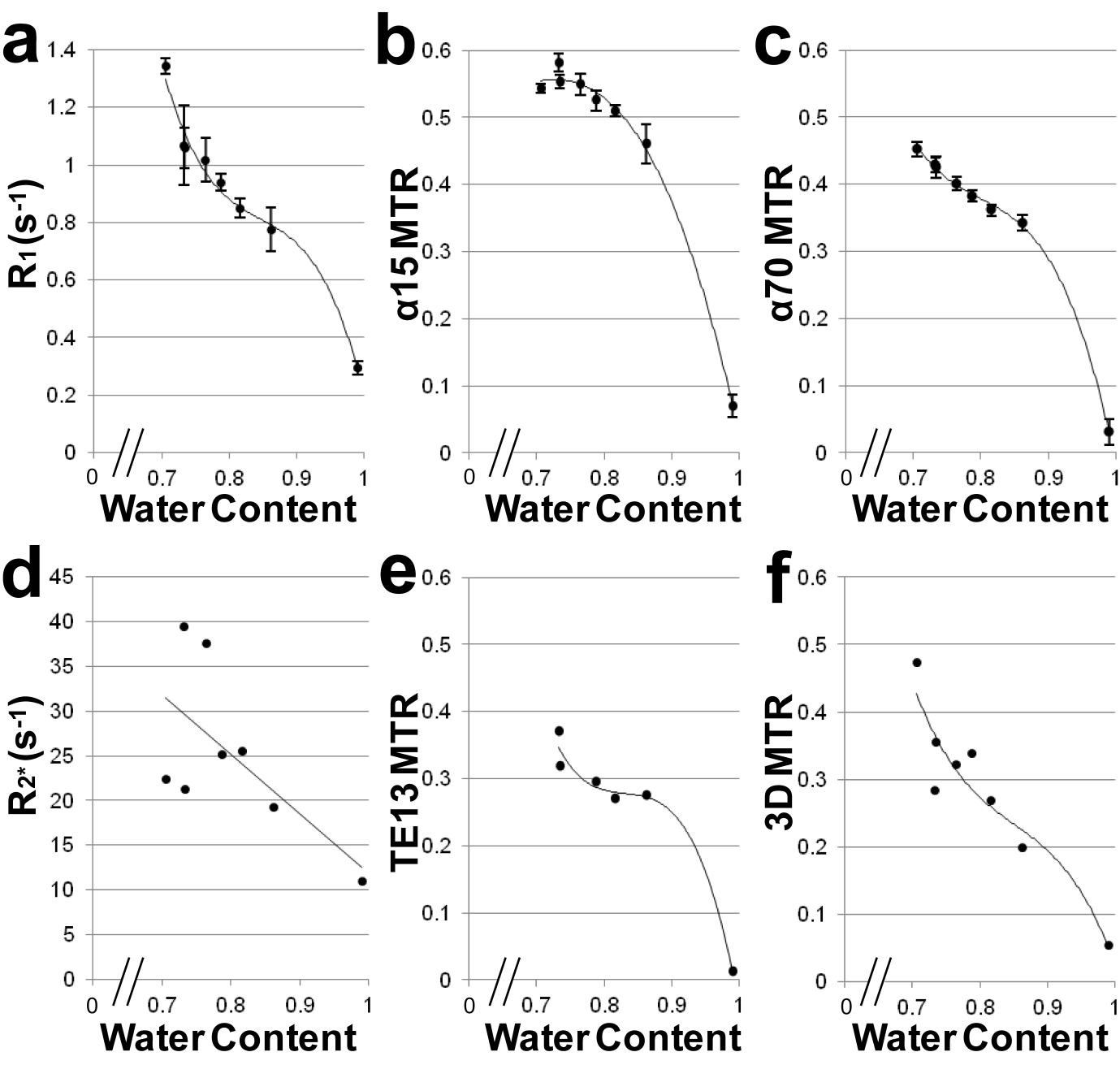


**Supplementary Figure 3** (a) R_1_, (b) magnetization-transfer ratio (2D FLASH, TR/TE = 863/4.4 ms, α = 15°), (c) magnetization-transfer ratio (2D FLASH, TR/TE = 863/4.4 ms, α = 70°), (d) R_2_*, (e) magnetization-transfer ratio (2D FLASH, TR/TE = 715/13.2 ms, α = 70°), and (f) magnetization-transfer ratio (3D FLASH, TR/TE = 47/7.5 ms, α = 22°) as a function of the water content in human brain. Equations and correlation coefficients are: (a) y = -113.29x^3^ + 287.52x^2^ + 244.5x + 70.47, r = -0.99, (b) y = -10.4x^3^ + 18.42x^2^ + 10.298x + 2.3039, r < -0.99, (c) y = -35.676x^3^ + 85.538x^2^ + 68.888x + 19.011, r < -0.99, (d) y = -66.351x + 78.25, r = -0.65, (e) y = -61.525x^3^ + 153.41x^2^ + 127.62x + 35.699, r = -0.99, (f) y = -30.301x^3^ + 77.288x^2^ + 66.429x + 19.466, r = -0.94.


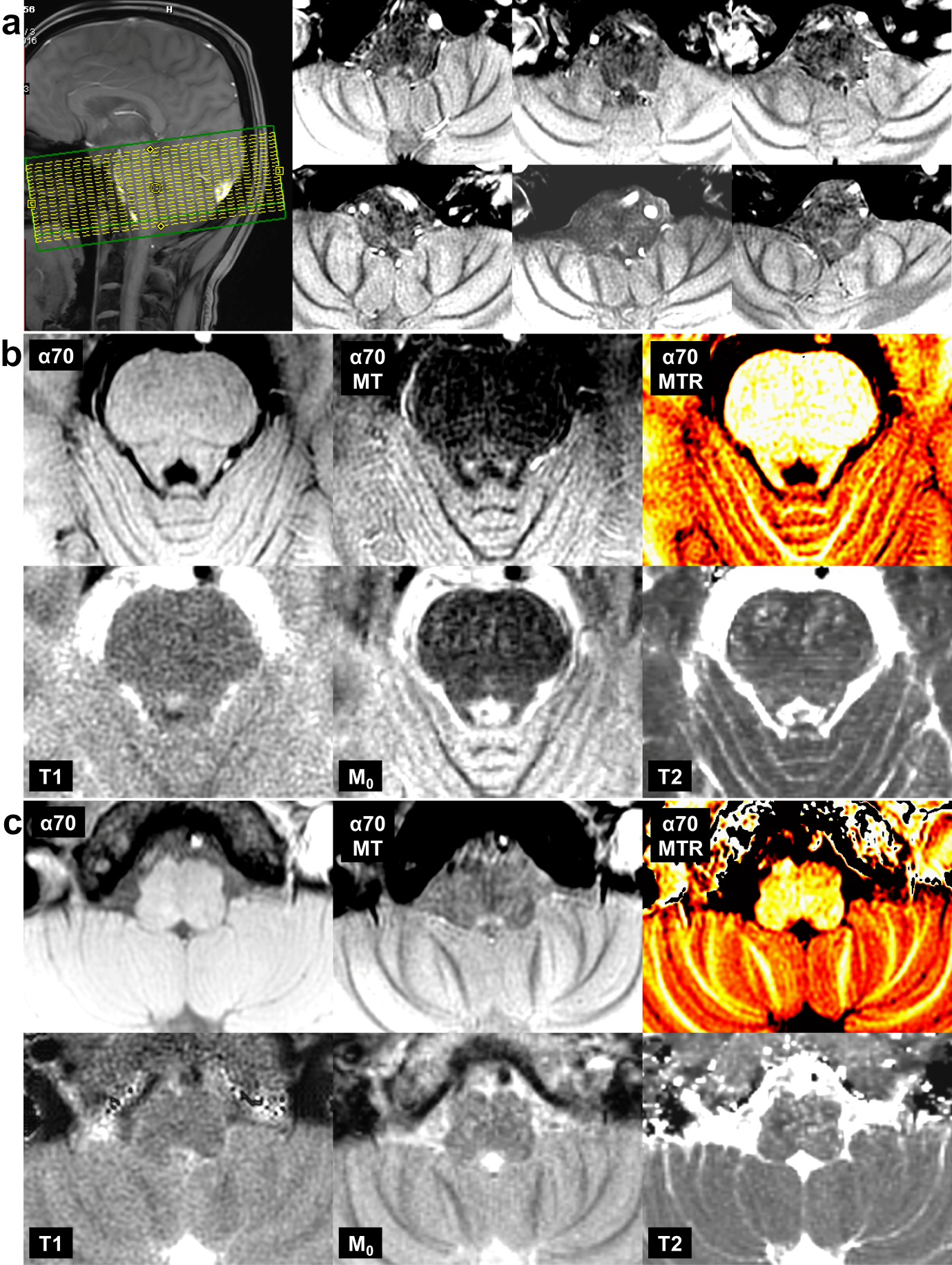


**Supplementary Figure 4** (a) (Left) Mid-sagittal human brain MRI illustrating the field-of-view selected for imaging the locus coeruleus and A2. (Right) transversal MRI of the A2 cell groups (arrows) of 6 different subjects. (b) transversal T_1_-weighted MRI of human brain without (α70) and with MT (α70MT), MT ratio map (α70MTR), T_1_ map (T1), M_0_ map (M_0_), and T_2_ map (T2) showing the locus coeruleus (arrows) and (c) the A2 cell group (arrows).

**Supplementary Table 1a** Signal-to-noise ratios (SNR) of the locus coeruleus (LC), A2 cell group, and brainstem as well as respective contrast-to-noise ratios (CNR) with constant off-resonance irradiation (200 Hz amplitude, 5000Hz offset) acquired at different on-resonance flip angles from 3~4-week-old female NMRI mice

| **Flip Angle (degree)** | | **10** | **15** | **18** | **22** | **26** | **30** |
| --- | --- | --- | --- | --- | --- | --- | --- |
|  | | *n* = 1 | *n* = 1 | *n* = 2 | *n* = 5 | *n* = 2 | *n* = 6 |
| **SNR** | **LC** | 26.6 | 30.2 | 31.9 ± 2.9 | 35.3 ± 1.2 | 27.2 ± 0.3 | 28.8 ± 5.5 |
|  | **A2** | 27.4 | 32.1 | 33.7 ± 3.5 | 35.8 ± 1.2 | 28.7 ± 0.3 | 29.6 ± 3.2 |
|  | **Brainstem** | 20.8 | 23.6 | 23.8 ± 0.6 | 25.6 ± 0.9 | 22.1 ± 0.6 | 23.6 ± 5.2 |
| **CNR** | **LC - Brainstem** | 5.8 | 6.6 | 8.1 ± 2.3 | 9.7 ± 1.1 | 5.1 ± 1.0 | 5.2 ± 1.0 |
|  | **A2 - Brainstem** | 6.7 | 8.5 | 9.9 ± 2.9 | 10.2 ± 1.1 | 6.7 ± 0.3 | 6.0 ± 3.0 |

Values are given as mean ± SD averaged across animals.

**Supplementary Table 1b** Signal-to-noise ratios (SNR) of the locus coeruleus (LC), A2 cell group, and brainstem as well as respective contrast-to-noise ratios (CNR) with constant off-resonance irradiation (100 Hz amplitude, 2500 Hz offset) acquired at different on-resonance flip angles from 3~4-week-old female NMRI mice

| **Flip Angle (degree)** | | **18** | **22** | **26** | **30** |
| --- | --- | --- | --- | --- | --- |
|  | | *n* = 5 | *n* = 6 | *n* = 6 | *n* = 6 |
| **SNR** | **LC** | 36.7 ± 5.2 | 36.7 ± 5.2 | 30.4 ± 4.4 | 27.5 ± 4.0 |
|  | **A2** | 39.0 ± 3.6 | 38.7 ± 4.7 | 31.6 ± 3.3 | 29.0 ± 3.4 |
|  | **Brainstem** | 27.7 ± 4.4 | 27.6 ± 4.0 | 24.2 ± 3.2 | 22.1 ± 3.4 |
| **CNR** | **LC - Brainstem** | 9.0 ± 1.1 | 9.1 ± 1.4 | 6.1 ± 1.9 | 5.4 ± 0.9 |
|  | **A2 - Brainstem** | 11.3 ± 1.0 | 11.1 ± 1.7 | 7.3 ± 0.5 | 6.9 ± 1.1 |

Values are given as mean ± SD averaged across animals.

**Supplementary Table 1c** Signal-to-noise ratios (SNR) of the locus coeruleus (LC), A2 cell group, and brainstem as well as respective contrast-to-noise ratios (CNR) with off-resonance irradiation of 200 Hz amplitude acquired at different frequency offsets from 3~4-week-old female NMRI mice

| **Offset (Hz)** | | **3000** | **4000** | **5000** | **6000** | **7000** |
| --- | --- | --- | --- | --- | --- | --- |
|  | | *n* = 3 | *n* = 5 | *n* = 5 | *n* = 5 | *n* = 2 |
| **SNR** | **LC** | 30.0 ± 3.4 | 32.7 ± 2.3 | 33.9 ± 2.2 | 35.1 ± 1.0 | 35.0 ± 2.4 |
|  | **A2** | 32.1 ± 3.4 | 33.7 ± 2.3 | 35.8 ± 1.3 | 35.7 ± 1.5 | 35.9 ± 1.9 |
|  | **Brainstem** | 20.9 ± 2.3 | 23.3 ± 1.6 | 24.5 ± 1.4 | 25.4 ± 1.2 | 26.1 ± 1.3 |
| **CNR** | **LC - Brainstem** | 9.1 ± 1.3 | 9.4 ± 1.0 | 9.4 ± 0.9 | 9.8 ± 1.2 | 9.9 ± 0.6 |
|  | **A2 - Brainstem** | 11.2 ± 1.8 | 10.3 ± 1.8 | 11.3 ± 0.7 | 10.3 ± 1.7 | 9.4 ± 0.8 |

Values are given as mean ± SD averaged across animals.

**Supplementary Table 1d** Signal-to-noise ratios (SNR) of the locus coeruleus (LC), A2 cell group, and brainstem as well as respective contrast-to-noise ratios (CNR) with off-resonance irradiation of 100 Hz amplitude acquired at different frequency offsets from 3~4-week-old female NMRI mice

| **Offset (Hz)** | | **2200** | **2500** | **2800** | **3100** |
| --- | --- | --- | --- | --- | --- |
|  | | *n* = 6 | *n* = 6 | *n* = 6 | *n* = 5 |
| **SNR** | **LC** | 37.7 ± 2.3 | 39.2 ± 1.8 | 40.9 ± 4.2 | 40.1 ± 1.2 |
|  | **A2** | 39.1 ± 1.6 | 39.6 ± 1.2 | 40.4 ± 3.2 | 41.1 ± 1.6 |
|  | **Brainstem** | 30.0 ± 1.8 | 29.6 ± 0.9 | 31.0 ± 1.8 | 31.4 ± 1.4 |
| **CNR** | **LC - Brainstem** | 7.7 ± 1.4 | 9.6 ± 1.4 | 9.9 ± 4.0 | 8.8 ± 0.9 |
|  | **A2 - Brainstem** | 9.2 ± 0.9 | 10.1 ± 0.9 | 9.5 ± 2.5 | 6.9 ± 0.4 |

Values are given as mean ± SD averaged across animals.

**Supplementary Table 1e** Signal-to-noise ratios (SNR) of the locus coeruleus (LC), A2 cell group, and the brainstem and respective contrast-to-noise ratios (CNR) with off-resonance irradiation of 5000 Hz offset acquired at different amplitudes from 3~4-week-old female NMRI mice

| **Amplitude (Hz)** | | **200** | **160** | **120** | **80** |
| --- | --- | --- | --- | --- | --- |
|  | | *n* = 4 | *n* = 4 | *n* = 3 | *n* = 2 |
| **SNR** | **LC** | 34.8 ± 2.0 | 36.0 ± 2.9 | 38.2 ± 3.1 | 39.8 ± 1.1 |
|  | **A2** | 35.7 ± 1.0 | 35.2 ± 1.2 | 38.0 ± 2.0 | 41.1 ± 0.6 |
|  | **Brainstem** | 25.4 ± 1.4 | 27.2 ± 1.9 | 30.4 ± 1.8 | 32.5 ± 3.1 |
| **CNR** | **LC - Brainstem** | 9.4 ± 1.4 | 8.8 ± 1.3 | 7.8 ± 2.3 | 7.3 ± 1.7 |
|  | **A2 - Brainstem** | 10.3 ± 1.5 | 8.0 ± 1.4 | 7.6 ± 1.6 | 8.6 ± 2.6 |

Values are given as mean ± SD averaged across animals.

**Supplementary Table 1f** Signal-to-noise ratios (SNR) of the locus coeruleus (LC), A2 cell group, and the brainstem and respective contrast-to-noise ratios (CNR) with off-resonance irradiation of 2500 Hz offset acquired at different amplitudes from 3~4-week-old female NMRI mice

| **Amplitude (Hz)** | | **100** | **75** | **50** |
| --- | --- | --- | --- | --- |
|  | | *n* = 5 | *n* = 6 | *n* = 7 |
| **SNR** | **LC** | 37.4 ± 2.3 | 38.1 ± 3.6 | 39.1 ± 4.1 |
|  | **A2** | 38.0 ± 1.6 | 38.3 ± 3.3 | 39.6 ± 4.1 |
|  | **Brainstem** | 28.2 ± 0.8 | 29.6 ± 1.9 | 32.1 ± 3.2 |
| **CNR** | **LC - Brainstem** | 9.2 ± 1.8 | 8.5 ± 2.1 | 7.0 ± 1.2 |
|  | **A2 - Brainstem** | 9.9 ± 1.0 | 8.7 ± 2.2 | 7.5 ± 1.3 |

Values are given as mean ± SD averaged across animals.

**Supplementary Table 1g** Magnetization transfer ratios (MTR) and signal-to-noise ratios (SNR) of the locus coeruleus (LC), A2 cell group, and the brainstem and respective contrast-to-noise ratios (CNR) acquired with two different 12 ms off-resonance irradiations from three-week-old female NMRI mice

| **Amplitude, Offset** | | **100 Hz, 2500 Hz** | **200 Hz, 5000 Hz** |
| --- | --- | --- | --- |
|  | | *n* = 7 | *n* = 7 |
| **MTR** | **LC** | 0.10 ± 0.03 | 0.24 ± 0.02 |
|  | **A2** | 0.11 ± 0.04 | 0.23 ± 0.02 |
|  | **Brainstem** | 0.20 ± 0.02 | 0.33 ± 0.01 |
| **SNR** | **LC** | 36.8 ± 2.1 | 35.6 ± 3.9 |
|  | **A2** | 37.5 ± 1.9 | 36.4 ± 2.8 |
|  | **Brainstem** | 27.9 ± 0.9 | 26.9 ± 2.4 |
| **CNR** | **LC - Brainstem** | 9.0 ± 2.2 | 8.7 ± 1.6 |
|  | **A2 - Brainstem** | 9.7 ± 1.1 | 9.5 ± 0.9 |

Values are given as mean ± SD averaged across animals.

**Supplementary Table 1h** Magnetization transfer ratios (MTR) and signal-to-noise ratios (SNR) of the locus coeruleus (LC), A2 cell group, and the brainstemas well as respective contrast-to-noise ratios (CNR) of three-week-old NMRI male and female mice

|  | | **female** | **male** |
| --- | --- | --- | --- |
|  | | *n* = 7 | *n* = 6 |
| **MTR** | **LC** | 0.24 ± 0.02 | 0.23 ± 0.02 |
|  | **A2** | 0.23 ± 0.02 | 0.22 ± 0.02 |
|  | **Brainstem** | 0.33 ± 0.01 | 0.33 ± 0.02 |
| **SNR** | **LC** | 35.6 ± 3.9 | 35.6 ± 2.6 |
|  | **A2** | 36.4 ± 2.8 | 36.4 ± 2.3 |
|  | **Brainstem** | 26.9 ± 2.4 | 26.5 ± 2.0 |
| **CNR** | **LC - Brainstem** | 8.7 ± 1.6 | 9.1 ± 0.7 |
|  | **A2 - Brainstem** | 9.5 ± 0.9 | 9.8 ± 0.7 |

Values are given as mean ± SD averaged across animals.

**Supplementary Table 2a** Magnetization-transfer ratios (2D FLASH, TR/TE = 863/4.4 ms, α = 70°), T_1_, and T_2_ of selected brain regions in human subjects (n = 6, 31.5 ± 7.5 y.o.)

| **Brain Regions** | **α70 MTR** | **T_1_ (s)** | **T_2_ (ms)** |
| --- | --- | --- | --- |
| **Frontal White Matter** | 0.45 ± 0.01 | 0.74 ± 0.02 | 68.1 ± 2.3 |
| **Prefrontal Cortex** | 0.34 ± 0.01 | 1.30 ± 0.13 | 96.0 ± 8.5 |
| **Caudate Nucleus** | 0.36 ± 0.008 | 1.18 ± 0.05 | 76.4 ± 2.0 |
| **Putamen** | 0.38 ± 0.008 | 1.06 ± 0.03 | 67.6 ± 2.7 |
| **Thalamus** | 0.43 ± 0.02 | 0.95 ± 0.06 | 66.5 ± 8.0 |
| **Globus Pallidus** | 0.43 ± 0.01 | 0.95 ± 0.12 | 64.7 ± 11 |
| **Red Nuclues** | 0.47 ± 0.009 | 0.83 ± 0.04 | 65.5 ± 3.3 |
| **Subthalamic Nucleus** | 0.45 ± 0.03 | 0.89 ± 0.05 | 60.3 ± 4.0 |
| **Substantia Nigra** | 0.40 ± 0.01 | 0.99 ± 0.08 | 67.9 ± 2.5 |
| **Locus Coeruleus** | 0.36 ± 0.008 | 1.18 ± 0.13 | 95.6 ± 4.2 |
| **Cerebrospinal Fluid** | 0.03 ± 0.02 | 3.41 ± 0.25 | 409.0 ± 1.0 |

**Supplementary Table 2b** Magnetization-transfer ratios (2D FLASH, TR/TE = 863/4.4 ms, α = 70°), T_1_, and T_2_ of noradrenergic neuron groups in human subjects (n = 6, 28.5 ± 2.3 y.o.)

| **Brain Regions** | **α70 MTR** | **T_1_ (s)** | **T_2_ (ms)** |
| --- | --- | --- | --- |
| **Locus Coeruleus** | 0.34 ± 0.02 | 1.02 ± 0.09 | 91.1 ± 9.6 |
| **A2** | 0.34 ± 0.03 | 1.16 ± 0.15 | 90.4 ± 10 |

**Supplementary Table 3** Magnetization transfer ratio (MTR), signal-to-noise ratio (SNR), and contrast-to-noise ratio in T_1_-weighted MRI before and 3 days after MnCl_2_

|  | | **Before Mn (*n* = 5)** | | **After Mn (*n* = 5)** | | |
| --- | --- | --- | --- | --- | --- | --- |
|  |  | MT (-) | MT (+) | MT (-) | MT (+) | |
| MTR | **Locus Coeruleus** | 0.26 ± 0.03 | | 0.22 ± 0.01 | | |
|  | Nerve Cell Assemblies | 0.28 ± 0.01 | | 0.22 ± 0.02 | | |
|  | Cerebral Cortex | 0.30 ± 0.01 | | 0.27 ± 0.03 | | |
|  | White Matter | 0.40 ± 0.01 | | 0.35 ± 0.02 | | |
| SNR | **Locus Coeruleus** | 47.1 ± 2.2 | 35.3 ± 2.4 | 63.7 ± 3.5 (+35%) | | 52.6 ± 4.5 **(+49%)** |
|  | Nerve Cell Assemblies | 43.8 ± 3.4 | 32.0 ± 2.7 | 67.0 ± 3.8 (+49%) | | 55.8 ± 4.9 **(+75%)** |
|  | a. Pyramidal Cell Layers | 43.4 ± 4.0 | 32.2 ± 2.7 | 70.3 ± 3.6 (+63%) | | 58.3 ± 4.9 **(+81%)** |
|  | b. Habenular Nuclei | 42.8 ± 2.6 | 31.3 ± 2.5 | 66.6 ± 5.2 (+56%) | | 56.0 ± 5.6 **(+79%)** |
|  | c. Purkinje Cell Layers | 45.2 ± 3.8 | 32.5 ± 3.1 | 64.1 ± 3.0 (+42%) | | 53.2 ± 4.3 **(+64%)** |
|  | Cerebral Cortex | 37.9 ± 3.2 | 26.7 ± 2.0 | 53.1 ± 3.7 (+37%) | | 42.3 ± 3.9 **(+50%)** |
|  | White Matter | 43.5 ± 2.9 | 26.5 ± 1.6 | 53.3 ± 1.7 (+23%) | | 36.9 ± 2.3 **(+39%)** |
| Contrast-to-noise ratio | | 0.24 ± 1.1 | 5.5 ± 1.3 | 13.7 ± 2.2 | | 19.0 ± 2.7 |

Values are given as mean ± SD, values in parentheses: % change from before Mn, MT = magnetization transfer, nerve cell assemblies = mean values of (a) the pyramidal cell layers of the hippocampus, (b) the habenular nuclei, and (c) the Purkinje cell layer of the cerebellum (see Supplementary Fig. 9), white matter = mean values of the corpus callosum and the cerebellar white matter, contrast-to-noise ratio = SNR (Nerve Cell Assemblies) – SNR (White Matter).
